# Self-patterning of rostral-caudal neuroectoderm requires dual role of Fgf signaling for localized Wnt antagonism

**DOI:** 10.1038/s41467-017-01105-2

**Published:** 2017-11-07

**Authors:** Nozomu Takata, Eriko Sakakura, Mototsugu Eiraku, Takeya Kasukawa, Yoshiki Sasai

**Affiliations:** 1 Laboratory for in vitro Histogenesis, RIKEN Center for Developmental Biology, 2-2-3 Minatojima-minamimachi, Chuo-ku, Kobe, Hyogo 650-0047 Japan; 2 Center for Vascular and Developmental Biology, Feinberg Cardiovascular Research Institute, Northwestern University, 303 East Superior Street, Chicago, IL 60611 USA; 3 Laboratory of Developmental Systems, Institute for Frontier Life and Medical Sciences, Kyoto University, 53 Kawahara-cho, Shogoin, Sakyo-ku, Kyoto, Kyoto, 606-8507 Japan; 4 Large Scale Data Managing Unit, RIKEN Center for Life Science Technologies, 1-7-22 Suehiro-cho, Tsurumi-ku, Yokohama, Kanagawa 230-0045 Japan; 5 Laboratory for Organogenesis and Neurogenesis, RIKEN Center for Developmental Biology, 2-2-3 Minatojima-minamimachi, Chuo-ku, Kobe, Hyogo 650-0047 Japan

## Abstract

The neuroectoderm is patterned along a rostral-caudal axis in response to localized factors in the embryo, but exactly how these factors act as positional information for this patterning is not yet fully understood. Here, using the self-organizing properties of mouse embryonic stem cell (ESC), we report that ESC-derived neuroectoderm self-generates a Six3^+^ rostral and a Irx3^+^ caudal bipolarized patterning. In this instance, localized Fgf signaling performs dual roles, as it regulates Six3^+^ rostral polarization at an earlier stage and promotes Wnt signaling at a later stage. The Wnt signaling components are differentially expressed in the polarized tissues, leading to genome-wide Irx3^+^ caudal-polarization signals. Surprisingly, differentially expressed Wnt agonists and antagonists have essential roles in orchestrating the formation of a balanced rostral-caudal neuroectoderm pattern. Together, our findings provide key processes for dynamic self-patterning and evidence that a temporally and locally regulated interaction between Fgf and Wnt signaling controls self-patterning in ESC-derived neuroectoderm.

## Abstract (web summary)

The neuroectoderm is patterned in a rostral-caudal axis in the embryo but how positional information is interpreted, is unclear. Here, the authors show that in mouse 3D embryonic stem cell cultures, there is self-patterning that depends on sequential activation of localised Fgf then Wnt signalling.

## Introduction

The mammalian central nervous system arises from epiblast-derived neural ectoderm^1^, which then forms a rostral-caudal (R-C) axial pattern that defines the location of the future forebrain, midbrain, hindbrain and spinal cord^2^.

At the cellular and tissue levels, it is thought that the processes of R-C neural axis (neuraxis) formation involves a number of differentiation and regionalization steps, including epiblast differentiation, the generation of three germ layers, neuroectoderm (or neural plate, NP) formation, and morphogen gradient-dependent specification of the embryonic neuraxis. However, our understanding of the links between these processes is still lacking, especially with regard to the intrinsic properties of neuroectoderm patterning along the R-C axis.

The pluripotent epiblast arises from the inner cell mass (or, when cultured in vitro, called embryonic stem cell [ESC]^3^) in the mammalian blastocyst^4^. The rostral region of the epiblast becomes ectoderm, one of three germ layers, which is subsequently resolved into non-neural and neural ectoderm (or neuroectoderm, NE)^1^. The NE is further destined for neural lineage and eventually regionalized along the embryonic axis to form the R-C patterned NE.

Peter Nieuwkoop’s “activation-transformation model” is a classical model of vertebrate R-C NE patterning^5, 6^. In this model, the ectoderm first receives an “activation” signal that neuralizes the ectoderm and induces its differentiation into forebrain^7^. Then, in the presumptive caudal region, a second “transforming” signal caudalizes the ectoderm’s regional identity^7^.

With regard to the neuralizing signal, it has been shown that the early ectoderm explants differentiate into SoxD^+^ neural cells when deprived of the influence of surrounding tissues^8^. In addition, in vitro reconstruction studies, which mimic the step-wise differentiation of ESC into epiblast and NE, show that mouse ectodermal cells also intrinsically adopt a neural lineage^9^, especially a rostral neural fate^10, 11^ (reviewed in ref. ^12^).

With regard to the transforming signal, it is widely accepted that the “Wingless” Wnt secreted family of ligands performs the role of the second caudalizing signal^13^. Indeed, in mice, *wnt1* expression is initiated and seen in the midbrain around embryonic day (E) 8.5^14^. Targeted disruption of *wnt1* has been shown to result in severe abnormalities in the caudal brain region^15, 16^. In this regard, Wnt1 has essential roles for caudal brain formation. Gain of function studies of Wnt ligands show that Wnt signaling has a strong capacity to caudalize NE in a dose-dependent manner^17, 18^.

Other molecules, such as “Fibroblast growth factors (Fgfs)”^19^, have also been shown to have a caudalizing action on the NE in vertebrates^20, 21^. In mice, exposing embryos to Fgf4 results in the lack of expression of forebrain markers and the expansion of caudal markers^22^. Another study demonstrated that the use of chick embryo Fgf4 and Fgf8 could induce Wnt1 expression^23^. Since the signal effects are thought to differ depending on timing, localization and concentration, exactly how Wnt and Fgf signaling-activity is temporally localized and results in the caudal formation has remained incompletely understood, especially during the early events of R-C NE development.

One challenge to studying the early events of mouse NE development is analyzing the formation of the patterned R-C NE at an early embryonic stage. It is difficult to visualize key steps and to isolate specific cell types in quantities large enough for genetic and chemical manipulation at distinct development stages. Moreover, due to the interplay of intrinsic and extrinsic signals, the developing embryo is a complex system. However, we have previously reported efficient methods for generating several parts of the NE in a three-dimensional (3-D) culture of reaggregated mouse ESCs in vitro^24–27^. One intriguing observation in the 3-D culture is the spontaneous formation of certain patterns within an aggregate of cells^28^. Although this culture begins with homogenous stem cell aggregates floating in a uniform culture environment, the resultant tissues exhibit non-uniform patterns with certain levels of structural order (reviewed in ref. ^29^). Furthermore, these tissues can self-form fairly complex structures, such as the optic cup, stratified cerebral cortex, and rathke’s pouch (even non-neural tissue)^27, 30, 31^. Thus, we believe this ESC 3-D culture system provides a useful model for investigating the intrinsic properties of early developing NE tissues and for purifying an essential signaling interplay.

The present study is designed to elucidate how intrinsic mechanisms drive the key processes of R-C NE patterning during neural tissue development. Here, using the self-organizing properties of ESCs, we show that ESC-derived tissue self-generates a Six3^+^ rostral and a Irx3^+^ caudal bipolarized patterning. By live imaging of multiple color knock-in reporter lines and genome-wide analysis, an initial rostral polarization is governed by localized Fgf signaling, which then induces Wnt signaling for a caudal polarization at later stage. Differentially expressed Wnt antagonists, Dkk1 and Sfrp1 perform roles in orchestrating the formation of a balanced rostral-caudal neural pattern. From our observation, we propose a model for self**-**patterning of the R-C NE that depends on sequential and localized activation of Fgf and Wnt signaling.

## Results

### A rostral-caudal neural pattern self-forms in ESC culture

When mouse ESCs were cultured in a chemically defined medium, the cells expressed a neural marker, *sox1*, around culture day 4 (Supplementary Fig. 1A, B). Flow cytometry analysis (using *Sox1::GFP* ESC^32^) showed that most of the cells were converted to Sox1::GFP^+^ cells by day 7 (Supplementary Fig. 1C), suggesting that the ESC-derived aggregates possessed NE character by culture day 7 in vitro.

**Fig. 1:**
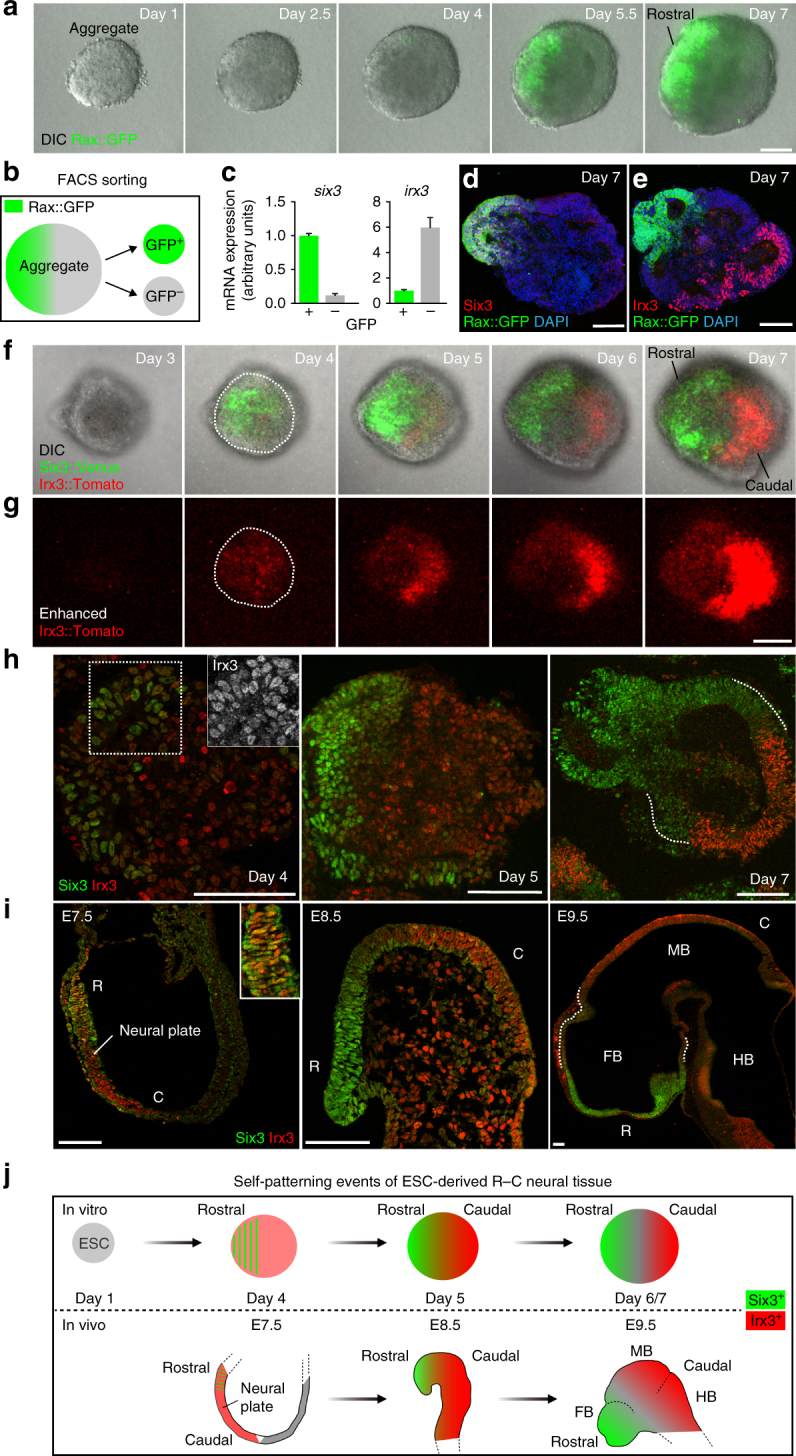
Self-patterning of rostral-caudal neuroectoderm in 3-D ESC culture. **a** Montage of images taken from Supplementary Movie 1, showing merged images of differential interference contrast (DIC) and Rax::GFP expression in the SFEBq aggregates, and epithelial structure formation around day 4. **b** Diagram of cell sorting of day-7 aggregates via fluorescence activated cell sorting (FACS) using the *Rax::GFP* ESC line. **c** Quantification of *six3* and *irx3* expression via RT-qPCR, following FACS sorting of GFP^+^ and GFP^−^ cells. Error bars indicate standard error of the mean (s.e.m) of each FACS-sorting experiment. Data represent mean ± s.e.m. **d**, **e** Immunohistochemistry was performed on cryosections of day-7 *Rax::GFP* aggregates using antibodies recognizing GFP, Six3 and Irx3. DAPI was used for counter staining. **f** Montage of images taken from Supplementary Movie 2, showing DIC, Six3::Venus and Irx3::Tomato expression. **g** Montage of images taken from Supplementary Movie 2, showing enhanced time-lapse images of Irx3::Tomato expression via ImageJ software to see a dim expression. Dotted lines in day-4 images show outline of aggregates (**f**, **g**). **h**, **i** Immunostaining of cryosectioned day-4, -5 and -7 aggregates (**h**); E7.5, 8.5 and 9.5 embryos (**i**), showing Six3 and Irx3 staining. R, rostral; C, caudal; E. embryonic day. A dotted square in day-4 aggregate (**h**) corresponds to a single channel image of Irx3 (white) shown via an inset. Dotted lines in day-7 aggregate (**h**) and E9.5 embryo (**i**) show the regions of weak Six3 expression. **j** Schematic diagram of in vitro and in vivo rostral-caudal NE formation. ESC, embryonic stem cell; FB, forebrain; MB, midbrain; HB, hindbrain. Scale bars, 100 µm (**a**, **d**, **e**, **g**, **h**, **i**). These images were one of *n* = 3 experiments (**a**, **d**–**i**)

A previous study showed that ESC-derived NE created by using this culture method goes on to express a rostral forebrain marker, Rax (also called Rx)^24^. Because of this, we used *Rax::GFP* ESC to monitor the formation of the rostral forebrain. Live imaging analysis showed that small patches of Rax::GFP^*+*^ cells first appeared in a speckled pattern (Fig. 1a and Supplementary Movie 1). By day 5, the Rax::GFP^*+*^ signals became noticeably stronger in one half of the aggregate (Fig. 1a), and this rostral-polarization trend continued through the next two days (Fig. 1a; see ratio of rostral-polarized pattern in Supplementary Fig. 1D).

To confirm the regional identity of day-7 NE, we next performed quantitative reverse transcription Polymerase Chain Reaction (RT-qPCR) analysis of fluorescence-activated cell sorting (FACS)-sorted cells (Fig. 1b). As previously reported^24^, Rax::GFP^*+*^ cells expressed another rostral marker, *six3* ^33^ (Fig. 1c). In contrast, Rax::GFP^−^—cells had high expression levels of *irx3*, a caudal marker^34^ (Fig. 1c). Moreover, Six3 expression was polarized to the same side of, and Irx3 to the opposite side of, the Rax::GFP^*+*^ region (Figs. 1d, e). These results indicate that ESC-derived day-7 NE possesses a Rax^*+*^/Six3^*+*^ rostral and Rax^−^/Irx3^*+*^ caudal bipolarized pattern as seen in in vivo R-C neuraxis formation.

To better observe the generation of this R-C NE patterning in vitro, we established a dual-fluorescence reporter ESC, a *Six3::Venus//Irx3::Tomato* knock-in line (Supplementary Fig. 1E-H). We observed generation of the R-C patterned NE (Fig. 1f, Supplementary Fig. 1I and Supplementary Movie 2; see Supplementary Fig. 1J for Rax expression in the Six3::Venus^+^ cells, and see also the ratio of Six3-Irx3 expression pattern in Supplementary Fig. 1K (there is some aggregate-to-aggregate variation in terms of Six3-Irx3 polarization)).

In order to compare in vitro aggregates with in vivo embryos, we performed immunostaining to detect endogenous Six3 and Irx3. Notably, day-4 aggregates showed Six3-localized and Irx3-globalized expression (Fig. 1h; see similar reporter expression in Fig. 1g and Supplementary Fig. 1I). Day-5 aggregates exhibited reciprocal patterning of Six3 and Irx3, as Irx3 decreased in the Six3-positive region (Fig. 1h). Day-7 aggregates showed Six3 expression in a decreasing gradient toward the Irx3^*+*^ boundary line (Fig. 1h). Dynamic Irx3 expression was observed by RT-qPCR analysis: *irx3* expression decreased from days 4 to 5 and then increased again by day 7, suggesting that caudal polarization occurs around day 5 (Supplementary Fig. 1L). At E7.5, Irx3 was expressed globally in the NP but Six3 was localized in the rostral NP, whereas Six3^*+*^ and Irx3^*+*^ cells occupied different regions in the NE at E8.5 (Fig. 1i and Supplementary Fig. 1M, N). E9.5 embryos showed a Six3 expression gradient decreasing toward the rostral end of the Irx3^*+*^ region (Fig. 1i).

To better characterize the regional identity of the aggregates, we performed immunostaining of Otx2, which is expressed in E9.5 NE from the forebrain to the midbrain^35^ and found that Otx2 expression was globally expressed within the day-7 aggregates (Supplementary Fig. 1O, P). Furthermore, Pax6, a forebrain marker^36^, was expressed in between the Rax::GFP^*+*^ and Irx3^*+*^ regions at day-7 (Supplementary Fig. 1Q, R). RT-qPCR data following FACS supported these marker expression patterns (Supplementary Fig. 1S).

These data indicate that by culture day 7, the R-C patterning self-forms within ESC-derived NE, which has similar regional identity to the in vivo E9.5 embryo at least from the forebrain to the midbrain regions, and developing aggregates exhibit dynamic expression of Six3 and Irx3 in a spatio-temporal manner. Notably, comparisons between in vivo embryos and in vitro aggregates showed similar dynamic regional patterning in regards to Six3 and Irx3 (Fig. 1j). Taken together, this culture system, which uses the self-organizing properties of ESCs, holds the potential to identify and understand the key processes for intrinsic R-C patterning formation, which would be difficult to do in the embryos.

### Fgf signaling is required for Fgf5^*+*^ epiblast differentiation

**Fig. 2:**
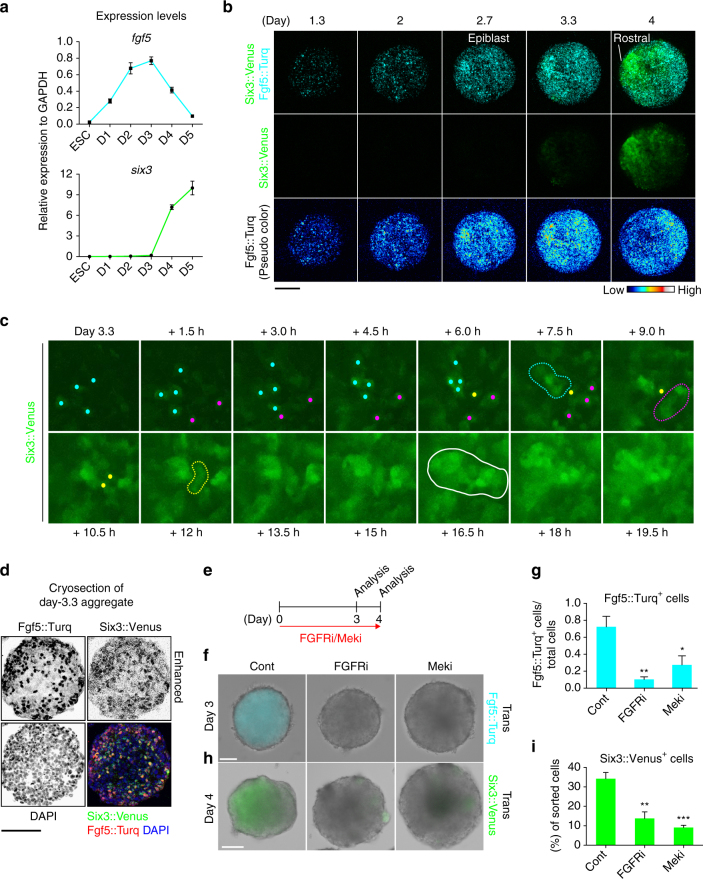
FGFR/Mek signaling is required for the differentiation of Fgf5^+^ epiblast-like cells. **a** Quantification of *fgf5* and *six3* expression via RT-qPCR from days 0 to 5 of embryonic stem cell (ESC) culture. D, day. **b** Montage of images taken from Supplementary Movie 3, showing upper panel; merged image of Six3::Venus and Fgf5::Turq (monomeric Turquoise2-HA-NLS), middle panel; Six3::Venus, lower panel; pseudo color of Fgf5::Turq. **c** Time-lapse imaging of Six3::Venus^*+*^ cells. Each color dot indicates a cell that is a component of each Six3^+^ small cluster. Broken lines (cyan, magenta and yellow) indicate each Six3^+^ small cluster. White solid line indicates a large cluster. **d** Immunohistochemistry against Venus and HA epitope (monomeric Turquoise2-HA-NLS) was performed on cryosections of day-3.3 aggregates, showing Six3::Venus and Fgf5::Turq signals. Six3::Venus signals were enhanced by the auto contrast function of ImageJ to see a dim expression. **e** Diagram of pharmacological inhibition of FGFR and Mek (FGFRi and Meki) by PD173074 and PD0325901, respectively. **f**, **h** Merged images of transillumination (Trans) and Fgf5::Turq (**f**) or Six3::Venus (**h**). **g** Quantification of Fgf5::Turq-HA-NLS positive cells via ImageJ analysis (See Methods). Total cells were counted by DAPI signals. **i** Quantification of Six3::Venus^+^ cells via FACS. Error bars indicate s.e.m of each experiment (**a**, **g**, **i**). Significance was determined using Dunnett’s test (**g**, **i**). *P < 0.05; **P < 0.01; ***P < 0.001. Cont, control. Scale bars, 100 µm (**b**, **d**, **f**, **h**). These images were one of *n* = 3 experiments (**b**, **c**, **d**, **f**, **h**)

To understand the key processes governing ESC-derived R-C patterning during earlier development periods, we investigated the events preceding the initial Six3^+^/Rax^+^ rostral polarization, which become evident by day 4 (Fig. 1a, f). To begin, we examined the expression of *fgf5*, due to its distinct in vivo role as an epiblast marker whose expression continues until the NP stage in the ectoderm of early embryos^37^. By quantifying *fgf5* expression during the days preceding Six3^+^/Rax^+^ rostral polarization, we found a striking increase in *fgf5* expression as the ESCs underwent differentiation, with peak expression detected at day 3, the time prior to initial rostral polarization (Fig. 2a). This *fgf5* expression dynamics was similar to a previous report^38^.

To simultaneously visualize Fgf5 and Six3 expression dynamics at the cellular level and in real time, we generated an *Fgf5::Turq* knock-in line (Supplementary Fig. 2A, B). We found that the Fgf5::Turq^+^ signals largely recapitulated the increase in *fgf5* (Fig. 2a), with peak fluorescence seen around day 3, a time period comparable to that seen in RT-qPCR data (Fig. 2a, b and Supplementary Movie 3). Importantly, the Six3::Venus^+^ region was polarized on the opposite side of the Fgf5::Turq^+^ region (Fig. 2b; see also the ratio of polarity regarding Six3 and Fgf5, and the average size of day-4 aggregates in Supplementary Fig. 2C, D).

We further sought to monitor Six3::Venus^*+*^ regions at the cellular level from day-3.3 aggregates, around the time when Six3::Venus^*+*^ expression starts. We found that Six3::Venus^*+*^ cells first appeared in a speckled manner, and then each proximally located Six3::Venus^*+*^ cell gathered to make a small cluster (Fig. 2c). Upon initial polarization, these rostral-cell clusters subsequently shared a spatially localized region, becoming a large cluster. Furthermore, during these localization processes, Six3::Venus^*+*^ signals became gradually stronger as a result of occupying the rostral region within the aggregate (Fig. 2c).

To better observe Fgf5 and Six3 expression, we performed immunostaining on cryosectioned day-3.3 aggregates. Fgf5 and Six3 were faintly expressed in the same cells, implying that initial Six3^*+*^ cells follow the Fgf5^*+*^ epiblast fate (Fig. 2d). A previous report suggested that Fgf stimulation is one of triggers for ESCs to enter a lineage commitment^39^, thus we performed pharmacological inhibition of Fgf signaling to test this. Inhibition of both Fgf Receptor (FGFR) and mitogen/extracellular signal-regulated kinase (Mek, a downstream mediator of Fgf signaling) from days 0 to 3 showed a remarkable reduction in the number of Fgf5::Turq^+^ cells (Fig. 2e, f, g). Furthermore, the number of Six3::Venus^+^ cells was also reduced by inhibiting FGFR/Mek (Fig. 2e, h, i). These results are consistent with the idea that Fgf5^+^ epiblast cells are the precursors for the Six3^+^ early neural cells.

We further analyzed Oct3/4, a pluripotent stem cell marker, which is expressed prior to Fgf5^40^. Consistent with the previous report^38^, *oct3/4* was expressed from ESC to day 3 and then subsequently decreased from day 3 to 5, the period during which *fgf5* expression also decreased (Fig. 2a and Supplementary Fig. 2E). Interestingly, we found that Oct3/4^+^ cells decreased at day 4 in the region where Six3::Venus^+^ signals were detected (Supplementary Fig. 2F, H). Also, Oct3/4^+^ cells coincided with Fgf5::Turq^+^ cells (Supplementary Fig. 2G, H; see also the expression pattern of day-3.3 aggregate in the Supplementary Fig. 2I). The reciprocal expression pattern of Oct3/4 and Six3 was also observed in the E7.5 embryo, suggesting that the temporal and spatial expression pattern of Six3 and Oct3/4 in the aggregates recapitulates the in vivo pattern (Supplementary Fig. 2J, K).

This implies that, through epiblast differentiation, the initial Six3^+^ rostral polarization seems to occur around days 3 to 4 in the culture resulting in the loss of Fgf5^+^ and Oct3/4^+^ expression in the rostral region.

### Localized Fgf signaling regulates Six3^+^ rostral polarization

**Fig. 3:**
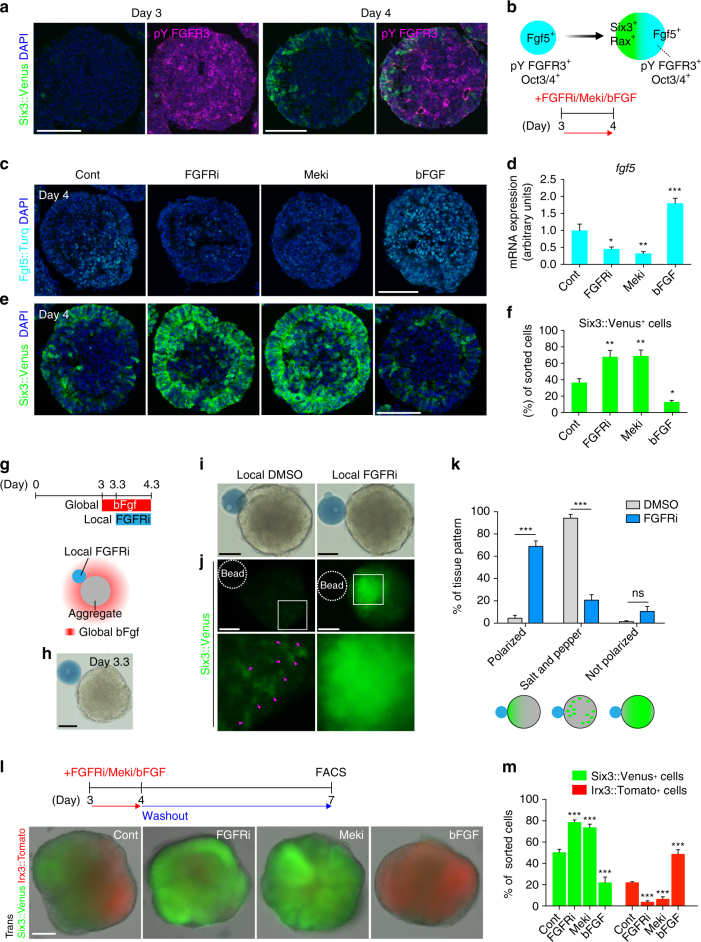
Six3^+^ rostral polarization is governed by Fgf/FGFR/Mek signaling. **a** Immunostaining using a phospho specific antibody against phospho (p)-tyrosine (Y) 724 FGFR3, showing pY724 FGFR3 with Six3::Venus signals in the day-3 and day-4 aggregates. **b** Schematics of gene expressions in day-3 and day-4 aggregates, and a timing of the addition of Fgf signaling antagonists (PD173074 and PD0325901) and agonist (bFGF). **c**, **e** Images of Fgf5::Turq and Six3::Venus signals in day-4 aggregates with pharmacological inhibition of FGFR/Mek and stimulation of Fgf signaling from culture day 3 to 4. **d** Quantification of *fgf5* expression via RT-qPCR. **f** Quantification of Six3::Venus^+^ cells via FACS. **g** Schematic of a local application experiment. Recombinant bFGF was used as a global application. **h** Bright field image of day-3.3 aggregate with a local bead. **i** Bright field images of day-4.3 aggregates with local beads soaked in either DMSO or FGFRi. **j** Six3::Venus fluorescent images of day-4.3 aggregates. Bottom panels are high magnification images of insets of upper images. Arrowheads (Magenta) indicate salt and pepper-like Six3^+^ cells in the DMSO condition where Six3::Venus^+^ signals are enhanced via the auto contrast function of ImageJ software to see a dim expression. **k** A graph shows the ratio of rostral polarization in the DMSO (*N* = 137) and FGFRi (*N* = 283) conditions. Polarized, Six3::Venus^+^ signals were polarized. Salt and pepper, Six3::Venus^+^ cells showed a salt and pepper pattern. Not polarized, Six3::Venus was globally expressed. **l** Schematic of the addition (only from day 3 to 4) and washout (at day 4) of PD173074, PD0325901 or bFGF. Below, merged images of transillumination (Trans) and fluorescence of day-7 aggregates in each condition. **m** Quantification of Six3::Venus^+^ and Irx3::Tomato^+^ cells via FACS. Error bars indicate s.e.m of each experiment (**d**, **f**, **k**, **m**). Significance was determined using Dunnett’s test (**d**, **f**, **m**) and unpaired *t*-test (**k**). *P < 0.05; **P < 0.01; ***P < 0.001. Cont, Control. Scale bars, 100 µm (**a**, **c**, **e**, **h**, **i**, **j**, **l**). These images were one of *n* = 3 experiments (**a**, **c**, **e**, **h**, **i**, **j**, **l**)

By day 4, the Fgf5::Turq^+^ signals were more pronounced on the presumptive caudal region opposite the Six3::Venus signals (Fig. 2b). This reciprocal expression pattern hinted at a role for Fgf signaling in the initial rostral polarization (Six3^+^/Fgf5^+^ bipolarity). Thus, we asked whether Fgf signaling acts in a polarized fashion within the aggregates during days 3 to 4, the time preceding rostral polarization. Interestingly, we found that phosphorylated tyrosine 724 residue of FGFR3 (pY724 FGFR3) signals were detected globally in the day-3 aggregate but only locally in the day-4 aggregate, suggesting that FGFR3 has a role in the initial rostral polarization (Fig. 3a). We also detected the local pY724 FGFR3 signals coinciding with Oct3/4 expression in the day-4 aggregate (Supplementary Fig. 3A).

To examine the role of Fgf signaling, we added PD173074, the inhibitor against FGFR3^41^ and Mek inhibitor at day 3, the time at which *fgf5* expression is at its maximum (Figs. 2a and 3b). At day 3, we found a reduction of Fgf5::Turq^+^ cells and *fgf5* expression (Fig. 3c, d; see also a reduction of Oct3/4 in Supplementary Fig. 3B) and a notable increase in the number of Six3::Venus^+^ cells (Fig. 3e, f). In the opposite experiment, the stimulation of day-3 aggregates with recombinant basic FGF (bFGF), in order to upregulate Fgf signaling, led to an increase in Fgf5::Turq^+^ cells and mRNA expression and a decrease in Six3::Venus^+^ signals (Fig. 3c–f). These lines of evidence imply that localized Fgf signaling, which are potentially mediated via FGFR3, prevents the presumptive caudal region from becoming the rostral region. In other words, the presumptive rostral region might be negatively regulated by Fgf signaling.

We also suspected other signaling pathways could have an influence on neural tissue, such as Sonic hedgehog (Shh), Bone morphogenetic proteins (Bmps)^42^ and Wnt signaling^43^. However, their inhibitors did not have a noticeable impact on Six3::Venus expression from days 3 to 4 (Supplementary Fig. 3C). Also, the inhibition of Wnt signaling did not affect the initial Six3 polarization, suggesting that Shh, BMP and Wnt are not main regulators of the initial rostral polarization (Supplementary Fig. 3D).

To further confirm the localized function of Fgf signaling on rostral polarization, we next performed a local manipulation of Fgf signal gradients. We pretreated day-3 aggregates with bFGF in a global manner and locally applied FGFRi-soaked beads to day-3.3 aggregates (Fig. 3g, h). We found the local FGFR inhibition locally induced the Six3^+^ polarization in the same side of the bead by 24 hr (Fig. 3i, j, k). Conversely, in a DMSO-soaked bead condition, the majority of pattern turned out to be a salt and pepper, suggesting that the rostral cluster process might be disrupted by the Fgf global application (Fig. 3i, j, k). The data from the local application experiment imply that the local FGFR inhibition protects a single rostral polarization (or clustering) from a salt and pepper state of rostral cells such that Fgf controls the initial rostral polarization.

We then asked whether Fgf signaling had a lasting effect on R-C polarity (or pattern). We exposed aggregates to either Fgf antagonists, FGFR/Mek inhibitors, or the agonist bFGF alone between days 3 and 4 (washout at day 4). When compared to day-7 control aggregates, which had an expected R-C pattern, aggregates treated with Fgf antagonists showed increased Six3::Venus expression but decreased Irx3::Tomato expression (Fig. 3l, m). In contrast, aggregates treated with bFGF showed decreased Six3::Venus expression and increased Irx3::Tomato expression (Fig. 3l, m). These data imply that the initial rostral polarization by Fgf/FGFR/Mek signaling is important for the establishment of later R-C patterning. Fgf signaling might have dual roles for the initial rostral polarization and maintaining presumptive caudal region.

### Wnt ligands differentially expresses in the caudal region

**Fig. 4:**
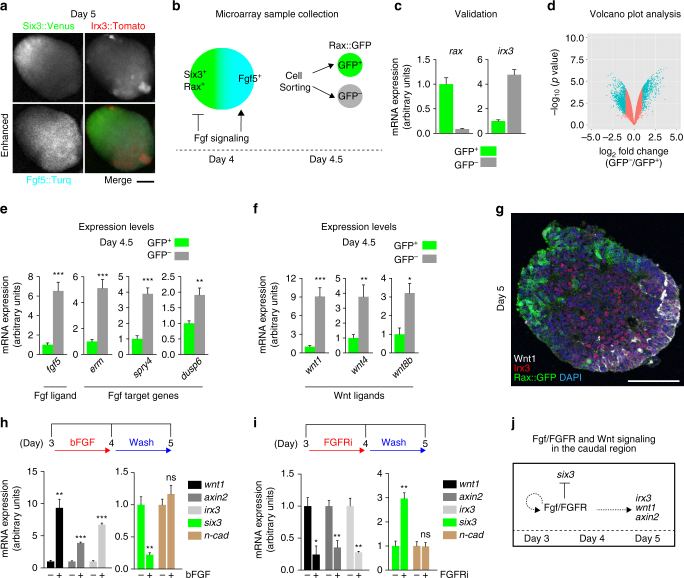
Genome-wide analysis of the polarized tissue reveals differential expression of Wnt signaling components in the caudal region. **a** Fluorescent images of a day-5 aggregate using a *Fgf5::Turq//Six3::Venus//Irx3::Tomato* ESC line. Fgf5::Turq signals were enhanced by the auto contrast function of ImageJ software due to its dim expression at culture day 5. **b** Schematic diagram of microarray sample collection. **c** A validation of microarray samples via RT-qPCR, showing *rax* and *irx3* expression in FACS-sorted GFP^+^ and GFP^−^ cells. **d** Volcano plot drawn by plotting points of all probe sets with log2 fold-changes of GFP^−^ expression values to GFP^+^ (X-axis) and *p*-values of significant differences (Y-axis). Blue dots indicate log2 fold-change of more than 1 (i.e., more than two-fold). **e**, **f** Quantification of Wnt and Fgf signaling-related-gene expressions via RT-qPCR, following FACS sorting of GFP^+^ and GFP^-^ cells. **g** Immunohistochemistry was performed on cryosections of day-5 *Rax::GFP* aggregates with antibodies recognizing Wnt1, Irx3 and GFP, showing Wnt1, Irx3 and Rax::GFP signals. **h**, **i** Quantification of gene expressions via RT-qPCR, following time-specific treatment of bFGF and PD173074. **j** Schematic of Fgf/FGFR and Wnt signaling relationship in ESC-derived caudal differentiation in vitro. Error bars indicate s.e.m of each experiment (**c**, **e**, **f**, **h**, **i**). Significance was determined using unpaired *t*-test. *P < 0.05; **P < 0.01; ***P < 0.001; ns, not significant (**e**, **f**, **h**, **i**). Scale bars, 100 µm (**a**, **g**). These images were one of *n* = 3 experiments (**a**, **g**)

We next sought to determine any potential signals that may establish the caudal region. We hypothesized that initial signals would be present at day 4.5, the period immediately preceding the appearance of the Irx3^+^ polarization in the presumptive caudal region (Supplementary Movie 2 and Fig. 4a). To screen for these signals, we examined the gene expression in day-4.5 rostral polarized aggregates using genome-wide microarrays following confirmation of the quality of samples between the groups (Fig. 4b, c).

We then performed microarray analysis in biological duplicate and found that 3367 genes were significantly changed between the GFP^+^ and GFP^−^ samples, as indicated by volcano plot and heat map analysis (Fig. 4d and Supplementary Fig. 4A). To systematically visualize global gene expression changes in these rostral and presumptive caudal cells, we utilized Ingenuity Pathway Analysis (IPA), a method that reveals relationships amongst genes^44^. As we expected, the Oct3/4 pathway was ranked in the top by the IPA and Oct3/4 was indeed expressed in the in vivo caudal NE, which also expressed Irx3 at the headfold stage (Supplementary Fig. 4B, C, D; headfold stage would be corresponding embryonic day to the day 4.5 of the culture). To better understand the characteristics of caudal and rostral cells, we compared this microarray result with a previous report^45^. Among the genes that were significantly changed in day-3 epiblast-like cells (vs. day-0 ESCs), we found that 1825 probe sets were significantly changed between caudal and rostral cells, as shown by two-dimensional volcano plot (Supplementary Data 1 for the common set of the significantly changed gene lists and Supplementary Fig. 4E, F). We also performed gene ontology analysis and found a couple of stem cell-related gene ontology biological processes (GOBP) in the enriched gene ontology items, further implying that a stem cell-related pathway is potentially involved in the initial rostral-caudal patterning (Supplementary Fig. 4G and Supplementary Datas 2, 3).

Interestingly, the Wnt/ß-catenin signaling pathway was identified as a 2^nd^ top pathway in the IPA analysis (Supplementary Fig. 4B). We first investigated whether the Wnt pathway plays a role in the caudal polarization of self-organizing NE. We then sought to determine any link between Fgf signaling (see Fig. 4a for weak Fgf5 expression in the caudal region) and the activation of the Wnt signaling pathway components.

We examined microarray data for differentially expressed components of the Wnt and Fgf signaling pathways and validated their expression. Consistent with our data, *fgf5* showed high expression in the GFP^−^ presumptive caudal region (Fig. 4e). In addition, the downstream Fgf signaling targets *erm*, *spry4*, and *dusp6* ^46^ showed an upregulation in GFP^−^ cells (Fig. 4e). In terms of Wnt ligands, canonical Wnt ligands *wnt1*, *wnt4*, *wnt8b* were significantly upregulated in GFP^−^ cells (Fig. 4f). We then sought to determine the boundaries of the *wnt1* expression region due to the fact that it is known to have a strong caudalizing role in NE in vivo. Notably, day-5 aggregates showed that the Wnt1^+^ region coincided well with the Irx3^+^ region, reminiscent of E8.5 embryos in which Wnt1 is expressed in the rostral end of the Irx3^+^ region (Fig. 4g and Supplementary Fig. 4H). Similar to previous reports showing Wnt target gene *axin2* expression in the caudal region^47^, Axin2 was expressed in the Irx3^+^ caudal region in the E8.5 embryo and day-5 aggregates, suggesting that canonical Wnt signaling is active in the Irx3^+^ caudal region (Supplementary Fig. 4I, J).

These results led us to ask whether Fgf signaling could influence the transcription of the Wnt signaling components. To address this question, we exogenously stimulated the aggregates with bFGF and assayed the expression of Wnt signaling components. Surprisingly, bFGF stimulation from days 3 to 4 led to a significant increase in the expression of *wnt1* and *axin2*, as well as a *six3* reduction and an *irx3* elevation but no significant change in a neural cadherin, *n-cadherin* (Fig. 4h). Conversely, the addition of FGFR inhibitor had the opposite effect of bFGF stimulation (Fig. 4i).

Taken together, these data suggest that Fgf signaling could regulate the transcriptional activation of the Wnt signaling components in the presumptive caudal region where both Fgf signaling (or feedback) and Wnt signaling could ensure further R-C bipolarization (Fig. 4j).

### A spatial distribution of Wnt agonists and antagonists

To further characterize the potential activation of the canonical Wnt signaling pathway during R-C pattern formation, we introduced a Wnt signaling reporter into the *Rax::GFP* line (Supplementary Fig. 5A).

To confirm the efficacy of the *Rax::GFP//7TCF::Cherry* reporter line, we utilized the canonical Wnt agonist CHIR99201 (CHIR). Live imaging showed that the application of agonist to ESCs and day-4 aggregates resulted in an activation of *7TCF::Cherry* within 12 h (Supplementary Fig. 5B and Supplementary Movie 4).

**Fig. 5:**
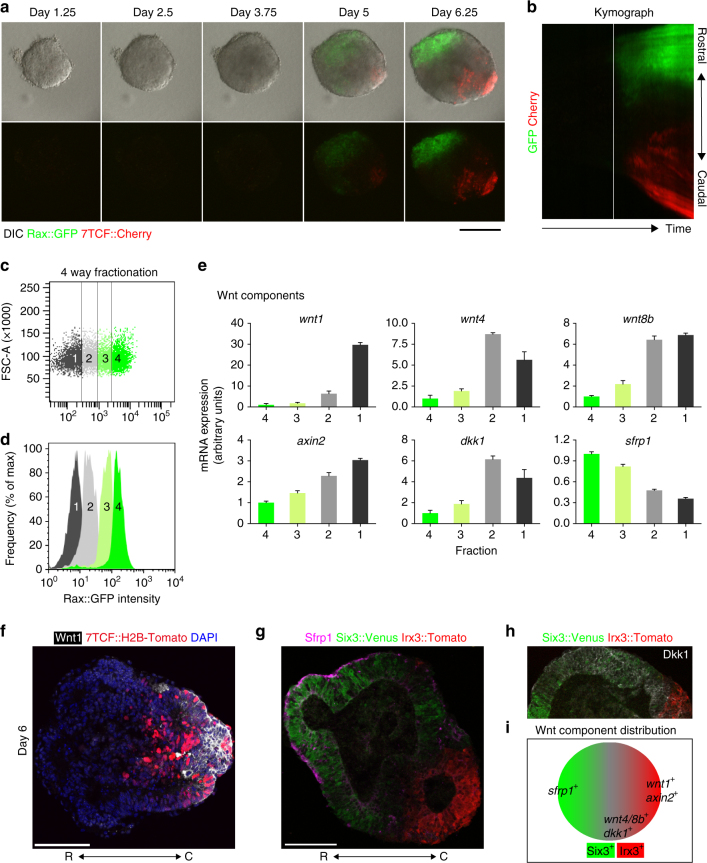
Wnt signaling components *wnt1/4/8b*, *sfrp1*, *dkk1* and *axin2* are spatially distributed along the R-C polarized neuroectoderm. **a** Montage of images taken from Supplementary Movie 5, showing upper panel; merged image of DIC (differential interference contrast), Rax::GFP and 7TCF::Cherry, lower panel; merged image of Rax::GFP and 7TCF::Cherry. **b** Kymograph analysis of time-lapse images by culture day 7, showing Rax::GFP and 7TCF::Cherry. White solid line indicates the time when Rax::GFP^+^ signals started to polarize. **c** Four way fractionation of day-6 cells by FACS, depending on Rax::GFP expression levels. **d** Confirmation of four fractions via FACS. **e** Quantification of Wnt signaling-related gene expression via RT-qPCR: *wnt1*, *wnt4*, *wnt8b, axin2, dkk1*, and *sfrp1*. Error bars indicate s.e.m of each FACS sorting experiment. **f**, **g**, **h** Immunohistochemistry was performed on cryosections of day-6 aggregates, showing Wnt1, 7TCF::H2B-Tomato, Sfrp1, Dkk1, Six3::Venus and Irx3::Tomato expressions. Dkk1 signals in Fig. 5h were also used to show in green in Supplementary Fig. 5J. **i** Schematic diagram of Wnt component distribution. R, rostral; C, caudal. Scale bars, 100 µm (**a**, **f**, **g**). These images were one of *n* = 3 experiments (**a**, **f**, **g**, **h**)

We then observed Rax::GFP//7TCF::Cherry^+^ fluorescence during the spontaneous formation of the R-C polarized NE; 7TCF::Cherry^*+*^ cells appeared by day 5 (Fig. 5a and Supplementary Movie 5). Notably, 7TCF::Cherry^*+*^ cells were located opposite to the Rax::GFP^*+*^ rostral region, further suggesting that canonical Wnt signaling helps to define the presumptive caudal region. Furthermore, kymograph analysis revealed that 7TCF:Cherry^*+*^ signals came later than rostral signals, indicating that canonical Wnt signaling is activated after rostral polarization (Fig. 5b).

We then asked whether the same Wnt signaling ligands identified in the microarray screen sustain the alternate regional expression in the day-7 aggregates. By FACS-sorting day-7 *Rax::GFP//7TCF::H2B-Tomato* cells into GFP^+^/RFP^−^ and GFP^−^/RFP^+^ fractions, we found that *wnt1*, *wnt4, wnt8b*, and *irx3* were expressed in GFP^−^/RFP^+^ cells, whereas *six3* was expressed in the GFP^+^/RFP^−^ cells (Supplementary Fig. 5C).

Because we observed the Six3 gradient at later development stage (culture days 6 to 7), we then suspected that Wnt signaling components would be involved in the formation of this gradient. To test whether Wnt signaling components are expressed as a gradient along the NE, we fractionated day-6 cells into four groups according to the level of Rax::GFP intensity (Fig. 5c, d). The levels of *six3* and *irx3* expression became gradually higher and lower in the GFP^+^ fractions, respectively (Supplementary Fig. 5D, E). Interestingly, five soluble Wnt components—*wnt1, wnt4, wnt8b, dickkopf1* (*dkk1*), *Secreted frizzled related protein 1* (*sfrp1*)—and the target gene, *axin2*, exhibited expression in a gradient (Fig. 5e). Specifically, *wnt1* expression became gradually higher in the GFP^−^ fractions (corresponding to the *irx3* ^+^ caudal region), whereas expression of *sfrp1*, whose gene product can bind to Wnt1 to downregulate Wnt signaling^48^, correlated well with GFP^+^ fractions (Fig. 5e). The expression pattern of Wnt1 and Sfrp1 at the protein level further supported the data observed in RT-qPCR (Fig. 5f, g; see the expression in the forebrain/midbrain regions of E9.5 embryos in Supplementary Fig. 5F, G, H). Additionally, Dkk1 protein was detected in between Six3::Venus^+^ and Irx3::Tomato^+^ regions, where Pax6 was also expressed (Fig. 5h and Supplementary Fig. 5I, J).

We also tested whether the Fgf signaling target genes identified in our microarray screen showed expression in a gradient manner. We found that *erm*, *spry4* and *dusp6* were expressed in the caudal fractions (fraction 1 and 2), implying Fgf signaling still acts to support the caudal identity (Supplementary Fig. 5K).

Together, these data confirm the activation of Wnt signaling around days 4.5 to 7, the time period in which the caudal polarization forms, and Wnt signaling agonists and antagonists show gradient expression along the NE (Fig. 5i).

### Wnt antagonism has a crucial role in R-C pattern formation

We then investigated whether Wnt signaling is required for R-C pattern formation. Before performing gain- and loss-of-function experiments, we endeavored to identify the appropriate time to manipulate Wnt signaling. Time course analysis of expression levels of Wnt signaling components showed that *sfrp1* and *wnt1* expression started to increase around day 4, prior to localized *TCF* reporter expression (Supplementary Fig. 6A; see also Supplementary Fig. 6B, showing a *sfrp1* biased expression in the day-3.5 rostral cells).

**Fig. 6:**
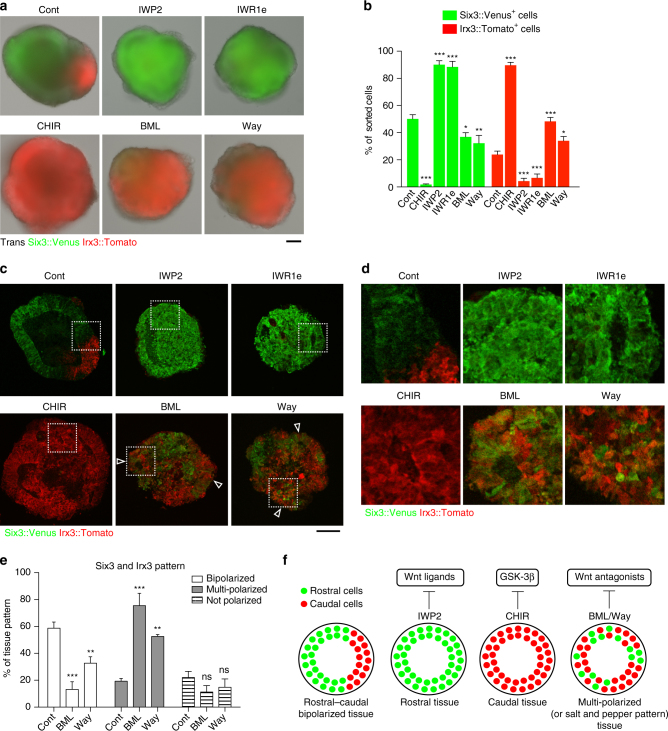
Wnt signaling antagonism has a crucial role for rostral-caural neuroectoderm bipolarization. **a** Merged image of day-7 aggregates, showing Trans (transillumination), Six3::Venus and Irx3::Tomato. Pharmacological inhibition was performed from day 4 to 7. Cont, Control; CHIR, CHIR99021; IWR1e, IWR-1-endo; BML, BML-287; Way, Way-262611. **b** Quantification of Six3::Venus^+^ and Irx3::Tomato^+^ cells via FACS. **c** Merged image of cryosectioned day-7 aggregates, showing the expression of Six3::Venus and Irx3::Tomato. Open white triangles indicate multi-polarized (or salt and pepper pattern) portions. **d** High-magnification images show the portion of broken-lined squares in **c**. **e** Quantification of the percentage of bipolarized and multi-polarized (including salt and pepper) patterns in Six3 and Irx3 expression. Cont (*N* = 53); BML (*N* = 44); Way (*N* = 36). **f** A diagram comparing the effect of inhibition of Wnt components on rostral-caudal patterning. Multi-polarization includes salt and pepper pattern. Error bars indicate s.e.m of each experiment. Significance was determined using Dunnett test (**b**, **e**). *P < 0.05; **P < 0.01; ***P < 0.001; ns, not significant. Cont, Control. Scale bars, 100 µm (**a**, **c**). These images were one of *n* = 3 experiments (**a**, **c**)

To analyze Wnt signaling’s function during R-C polarity formation, we used several chemical inhibitors against Wnt signaling components (Supplementary Fig. 6C). When Wnt agonist CHIR was added to the culture during days 4 to 7, we observed a dramatic decrease in Six3::Venus signals and an increase in Irx3::Tomato signals (Fig. 6a, b). A previous study reported that Wnt activation during ESC differentiation promotes neuromesoderm fate as seen in the neuromesoderm of E8.5 embryo, leading to a spinal cord identity^49^. For this reason, we suspected that the addition of the Wnt agonist induces the spinal cord identity. Via immunostaining of Sox2 and Brachyury, which are known to be expressed in the neuromesodermal progenitor cells^50^, we could not detect any obvious double positive cells, suggesting that activation of Wnt signaling does not induce neuromesoderm (or spinal cord)-like cells, at least during the assay period (Supplementary Fig. 6D, E). In contrast to Wnt activation, when Wnt inhibitors IWP2 and IWR1e were added, Six3::Venus expression substantially increased, whereas Irx3::Tomato expression substantially decreased (Fig. 6a, b). These results suggest that canonical Wnt signaling does indeed play a major role in R-C polarity at the later stage.

We next sought to analyze the function of the Wnt antagonists Sfrp1 and Dkk1. To test this, we treated ESC-aggregates with BML-287 an inhibitor of Sfrp1 and Way-262611 a Dkk1 inhibitor during days 4 to 7. Interestingly, we found that Six3::Venus expression partially decreased while Irx3::Tomato expression partially increased with the application of these chemical inhibitor (Fig. 6a, b), implying that Sfrp1 and Dkk1 have a certain role in the rostral- and caudal-regulation.

To further examine the effect of these chemical inhibitors at the cellular and spatial levels, we performed immunostaining and found that Wnt activation by CHIR led to an increase in caudalization (Fig. 6c, d). By contrast, Wnt inhibition by IWP2 and IWR1e led to an increase in rostralization, even at the cellular level (Fig. 6c, d). Importantly, Sfrp1 and Dkk1 inhibition showed multi-polarization of the rostral and caudal cells, including salt and pepper pattern (Fig. 6d). We also quantified the results from Wnt antagonist inhibition and found that both BML-287 and Way-262611 significantly decreased the ratio of R-C bipolarization but increased multi-polarization (Fig. 6e).

To confirm the role of Sfrp1 and Dkk1, we generated transgenic lines harboring short hairpin expression cassettes against *sfrp1* or *dkk1*. The addition of Dox specifically decreased *sfrp1* and *dkk1* mRNA in the sh[*sfrp1*] and sh[*dkk1*] ESCs, respectively (Supplementary Fig. 6F). Furthermore, knockdown of *sfrp1* and *dkk1* decreased the ratio of bipolarization but increased multi-polarization of rostral and caudal cells, including salt and pepper pattern (Supplementary Fig. 6G). Together, these data indicate that the differential Wnt signaling components are required for the establishment of the self-patterned R-C NE (Fig. 6f). In potentia, Wnt antagonists, Sfrp1 and Dkk1 would ensure rostral and caudal bipolarization in the extracellular space by locking the polarity and protecting it from environmental noise (Fig. 6f).

## Discussion

There are two reports regarding early tissue polarization from mouse ESCs in homogenous culture conditions. One is a pioneering report that shows ESCs have an ability to intrinsically generate an axis-like polarity, which demarcates the Brachury^+^ region via Wnt signaling^51^. Another describes “gastruloids” that exhibit a process relevant to gastrulation even in in vitro conditions^52^. A more recent report showed that BMP4-supplemented homogeneous conditions generate size- and geometry-dependent spatial patterning in human-ESC self-organization culture. However, human-ESC differentiation begins from a primed-state (or epiblast-like state), whereas mouse ESC differentiation begins from a naïve-state (or ICM-like state)^53–55^. Thus, ESC culture systems serve as good models for exploring the signaling cues which govern intrinsic tissue patterning in the minimal condition in vitro. We here suggest that in a chemically defined NE-induction culture, ESC-derived aggregates have a striking ability to form an R-C pattern, likely via intrinsic molecular gradient information. Indeed, our data shows that Fgf and Wnt signaling-mediated spatiotemporal events may provide a better understanding of tissue intrinsic R-C NE development and lead to focusing on the aspect of self-patterning ability of neural tissue (demarcated by Six3 and Irx3 expression patterns) in related fields.

**Fig. 7:**
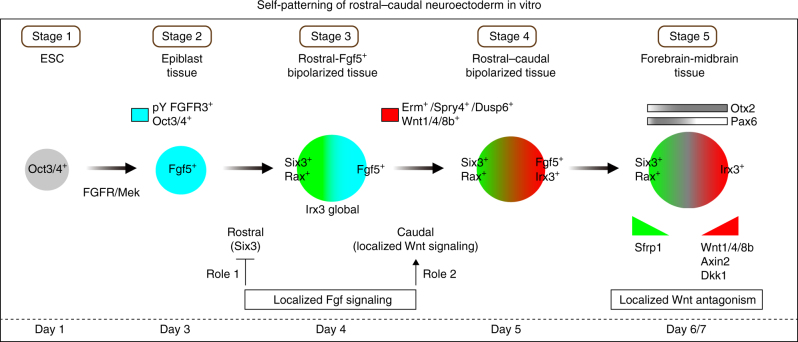
Overview of Fgf and Wnt signaling in rostral-caudal patterning of ESC-derived neuroectoderm. The processes of self-patterning in 3-D ESC culture. ESC differentiates into Fgf5^+^ epiblast. Six3^+^ rostral polarization is spontaneously generated. Localized Fgf signaling performs dual roles; in the early stage, suppression of Six3^+^ rostral cells in the presumptive caudal region; in the later stage, promotion of Wnt signaling. Localized Wnt signaling generates a Irx3^+^ caudal identity. Wnt antagonist and agonist gradients maintain a rostral-caudal neural pattern, which would be similar to the region from forebrain to midbrain in vivo

Based on our observations, we group the ‘self-patterning events’ into five distinct stages (Fig. 7). Namely, the Oct3/4^+^ ESC aggregate (Stage 1) differentiates into Fgf5^+^ epiblast-like tissue through FGFR/Mek signaling (Stage 2). Subsequently, Six3^+^/Rax^+^ rostral cells emerge and polarize in the region opposite to the localized Fgf5^+^ region, in which FGFR/Mek activity continues to repress Six3^+^ rostral polarization (Stage 3; Irx3 is expressed globally). Through Fgf-induced upregulation of Wnt signaling, Irx3^+^ cells localize in the region opposite to the Six3^+^/Rax^+^ region to become R-C bipolarized tissue (Stage 4). By stage 5, the forebrain to midbrain-like tissue that regionally expresses Otx2 and Pax6, reminiscent of its in vivo counterparts, is generated. The differential expressions of Wnt ligands and antagonists act to prevent NE from becoming a non-polarized and a multi-polarized pattern, respectively. In this manner, sequentially patterned R-C fate and localized signaling would be necessary to acquire the spontaneous NE patterning. In our previous ESC cultures to induce 3-D complex tissues, the controlling Fgf and Wnt signaling is also crucial at different times and dosages. Additionally, our self-organization culture exhibits essential aspects of multicellular system, highlighting self-assembly, self-patterning and self-driven morphogenesis from a homogeneous culture condition^29^. Of particular importance is self-patterning that initially seems to follow history-dependent rules to build minimal patterns, which can stabilize their fate in a sequential order. Notably, an initial neural pattern (or a minimal neural pattern) occurs from stage 2 to 3 when Six3^+^ rostral cells start to appear and be polarized. An interesting observation is that Six3^+^ rostral cell cluster formation might be under the control of a self-assembly mechanism, perhaps making the minimal pattern more robust.

As Six3, the earliest known rostral NE marker, is the key player in early rostral NE development, its main function is to repress Wnt1 expression, thus preventing differentiation of rostral from caudal NE in mouse^33, 56^. In this regard, the monitoring and analysis of Six3 expression (or Six3 positive cells) from early NE development onward would be quite essential to understanding not only rostral formation but also caudal regulation. Our current study successfully added additional information on rostral NE formation by combining genome-editing technology and ESC culture. The initial Six3^+^ rostral polarization was negatively regulated by FGFR/Mek signaling (Role 1 of Fgf signaling) while the inhibition of Wnt secretion or the canonical Wnt pathway had less of an effect on Six3 expression and polarization (Supplementary Fig. 3C, D). In addition, *wnt1* expression was evident in mRNA levels from culture day 4 and later (Supplementary Fig. 6A). Also, Wnt reporter signals come later after an initial rostral appearance (Fig. 5b). Therefore, FGFR/Mek signaling’s effect on rostral polarization is considered to be earlier than Wnt signaling’s effect. Importantly, time-lapse imaging and chemical inhibition analysis suggest that the Fgf5^+^ region maintains Fgf signaling. In fact, initial polarity driven by Fgf signaling has a lasting effect on later R-C patterning (Fig. 3l, m). A previous study has shown that the knockout of one of the Fgf ligands, Fgf8, caused abnormally expanded Six3 expression to the caudal ectoderm, raising the possibility that Fgf8 regulates rostral gene expression in in vivo NE^57^. Although the identification of Fgf ligands for FGFR/Mek signaling in this context still remains unknown, we hinted that FGFR3 was active in the Fgf5^+^ region of rostral-polarized tissue and acts as one of the receptors for Fgf ligands. Additionally, our microarray data will help to identify other key ligands.

One of our interesting observations is that the Fgf5^+^ presumptive caudal region was positive for Oct3/4, a key regulator of stem cell pluripotency, where active FGFR3 signals were detected. In the NE of embryos, Oct3/4 is locally expressed in the presumptive caudal region (opposite side to Six3^+^ region) from NP to headfold stage. From our microarray analysis, the Oct3/4 pathway was significantly active in the caudal samples whose RT-qPCR data showed higher Fgf-target gene expression. Thus, it is now necessary to consider the pluripotent pathway coupled-Fgf signaling, which would regulate FGFR3 upon caudal NE polarization.

The key event for the caudal polarization demonstrated by this study is Wnt regulation. Our series of analyses indicate that Fgf signaling in the presumptive caudal region upregulates Wnt1 expression, leading to Irx3 caudal gene expression (Role 2 of Fgf signaling). This regulation could then make the NE a R-C bipolarized pattern. Subsequently, Wnt signaling components are distributed in a gradient manner to generate Wnt antagonism by stage 5 (Fig. 7).

We were intrigued by the question of how R-C polarity is sustained. We showed that chemical- and RNAi-mediated disruption of *sfrp1* and *dkk1* caused multi-polarized (or salt and pepper) pattern in the localization of rostral and caudal cells. This result would be one of answer demonstrating that these two Wnt antagonists indeed regulate R-C polarity formation in a tissue-intrinsic manner. From that, one possible mechanism that could explain this is simple downregulation of Wnt signaling by Sfrp1 and Dkk1 antagonism. However, there are still remaining questions as to how Sfrp1 and Dkk1 expression is spatiotemporally regulated within the NE and how these two molecules modulate Wnt signaling in intrinsic R-C polarity formation.

Regarding Sfrp1, Wnt ligands normally suppress the rostral fate, meaning that transcriptional *sfrp1* expression is restricted by Wnt-ligand activity. In turn, extracellularly secreted Sfrp1 may suppress Wnt ligand activity at the protein level. Although vertebrates’ Sfrp1 can bind to Wnt1, Wnt4 and Wnt8 to attenuate Wnt signaling in the ligand level^48, 58, 59^, there still remains the question of how Sfrp1 binds to the Wnts within the ESC-derived neural-tissue scale. In a similar fashion to how, as a previous report showed, Wnt1, Wnt4 and Wnt8b are expressed at the different times and in different regions of the developing vertebrate brain^60^, Sfrp1 might interact with Wnt1, Wnt4 and Wnt8b to regulate their functions at specific times and in specific regions. Although *sfrp1* is preferentially expressed even in day-3.5 early Six3^+^ rostral cells, future studies are necessary in order to explore the biochemical binding of Sfrp1 to Wnt1, Wnt4 and Wnt8b, as well as the molecular function of these Wnt ligands over time during NE development (Supplementary Fig. 6B). Additionally, *dkk1*, one of the Wnt target-genes^61^, could inhibit Wnt signaling at the receptor level. The localization of Dkk1 expression seen in this study seems to be in a similar pattern to *wnt4*, likely reflecting the Wnt4-mediated Wnt signaling at stage 5 in ESC culture (Fig. 5e, h). However, since the inhibition of Sfrp1 and Dkk1 has a partial effect on the expression of rostral and caudal genes, we could not exclude the possibility that other Sfrp1 and Dkk family members are also involved in R-C gene regulation.

Regarding another functional aspect of antagonists, in Xenopus the Sfrp1 family has both an antagonistic role and an expansionary role, in that it binds to modulate the Wnt ligand diffusion rate, resulting in an expansion of the Wnt signaling region^62^. Notably, we observed that Wnt ligands and Sfrp1 are expressed in the same region (see fraction 2 and 3 in Fig. 5e). In this manner, these molecules might potentially interact in the extracellular space to modulate the Wnt ligand location and activity in ESC-derived aggregates. Considered collectively, future efforts to employ the visualization of Wnt ligands and their inhibitors in the living condition at different time points may offer further support for Wnt antagonism during NE development.

In conclusion, whereas extrinsic controls usually coexist with self-patterning in vivo and cooperatively support the R-C pattern formation, ESC-derived tissues in vitro have certain amplification systems which utilize relatively minimum localized signals (or simple local triggers) in order to ensure proper patterning. However, a comparison of mouse ESC (naive) with human ESC (primed) cultured in a self-organizing culture might assist in answering these key remaining questions: “what is a trigger?” and “how does the initial pattern arise from minimum culture condition?”.

## Methods

### ESC culture

Mouse ESC; *EB5* (RIKEN BioResource Center, Cell Number: AES0151)^63, 64^, *Sox1::eGFP* (*GFP*) (a gift from Austin Smith)^32^, *Rax::eGFP* (*GFP*) (RIKEN BioResource Center, Cell Number: AES0145)^24^ cells were maintained and SFEBq culture (serum-free floating culture of embryoid body-like aggregates with quick reaggregation) was performed^24^. Briefly, SFEBq begins with the quick reaggregation of dissociated mouse ESCs (3000 cells/aggregate) in each well of a 96-well plate (low cell-adhesive coating) and in a growth factor–free chemically defined medium (gfCDM), which is free of knockout serum replacement and other growth factors. Trypan blue staining was used to exclude died cells upon counting cells for accurately seeding 3000 cells. Mycoplasma contamination was routinely tested for in each ESC line.

### Immunostaining

For whole mount immunostaining, Slc:ICR mice were purchased from Japan SLC, Inc. Embryos were dissected in PBS and routinely fixed with 4% paraformaldehyde/PBS for 30 min at room temperature (RT). Fixed embryos were washed three times with PBS and immersed in PBS containing 1% Triton X-100 for 1 h at RT, and then blocked in blocking buffer (2% skim milk in PBS) for 2 h at RT. The embryos were incubated overnight at 4 °C with primary antibodies that were diluted with the blocking buffer at an appropriate concentration: Sox1: (rabbit, 1:200, Cell Signaling Technology, #4194), Six3: (rabbit, 1:1000, TaKaRa custom AS3014 and AS3984), Irx3: (Guinea pig, 1:1000, TaKaRa custom MS870G). After washes with 0.05% Tween/PBS for 1 h at RT, the embryos were incubated for 6 h at 4 °C with DAPI as a counter staining and appropriate secondary antibodies conjugated with the fluorescent probes, Alexa Fluor-488 (1:1000, Molecular Probes) or Cy3/Cy5 (1:200, Jackson ImmunoResearch) and then washed with 0.05% Tween/PBS for 1 h at RT. Immunostained embryos in PBS were mounted on glass bottom dishes for image analysis. For cryosectioned samples, imunostaining was performed by standard protocols^24^. Antibodies were used as follows: GFP (rat, 1:500, NACALAI, 04404-84), GFP (mouse, 1:500, Molecular probes, A11120), Six3 (rabbit, 1:1000, TaKaRa custom AS3014 and AS3984), Irx3 (Guinea pig, 1:1000, TaKaRa custom MS870G), Living Colors®DsRed (rabbit, 1:500, Clontech 632496), Rax (rabbit, 1:1000, TaKaRa custum PU42216BS and Guinea pig, 1:1000, TaKaRa custum MS8407-3), Otx2 (rabbit, 1:1000, Abcam ab21990), Pax6 (mouse, 1/500, R&D, MAB1260), HA epitope (mouse, 1:1000, Covance, MMS-101R), Oct3/4 (mouse, 1/200, BD, 611202; Only for this antibody, 5% Donkey serum was used in PBS as a blocking purpose), phospho Y724 FGFR3 (rabbit, 1/250, Abcam, ab155960), Wnt1 (rabbit, 1:500, Abcam, ab15251), Axin2 (rabbit, 1/2500, Abcam, ab32197), Sfrp1 (rabbit, 1:500, Abcam, ab126613), Dkk1 (rabbit, 1/500, Abcam ab61034), Sox2 (goat, 1/250, Santa cruz, sc-17320), Brachyury (goat, 1/1000, R&D, AF2085). Images were taken with a Zeiss LSM 710 or 780 confocal laser-scanning microscope.

### RNA extraction, cDNA synthesis and RT-PCR

Total RNA was extracted using QIAcubu’s RNA micro extraction mode (Qiagen), and cDNA was synthesized using SuperScript II Reverse Transcriptase (Invitrogen). RT-qPCR reactions were performed using a 7500 Fast Real-Time PCR System and SYBR Green (Applied Biosystems) with primers, which target exon-exon boundaries designed by Universal Probe Library Assay Design Center (Roche). All processes were based on the manufacturer’s instructions and the expression level of each mRNA was normalized to GAPDH. Data were displayed as the arbitrary units or as the relative values to each control. Standard curves were estimated using a series of dilutions of cDNA purified from mouse embryos and ESC-derived aggregates. Primers used were as follows: *sox1*, forward 5′-CCTCGGATCTCTGGTCAAGT-3′, reverse 5′-GCAGGTACATGCTGATCATCTC-3′; *six3*, forward 5′-CCGGAAGAGTTGTCCATGTTC-3′, reverse 5′-CGACTCGTGTTTGTTGATGGC-3′; *irx3*, forward 5′-CAACGAGCACCGCAAGAA-3′, reverse 5′-TGGTGATGATGGCCAACATG-3′; *fgf5*, forward 5′-GCGATCCACAGAACTGAAAA-3′, reverse 5′-ACTGCTTGAACCTGGGTAGG-3′; *otx2*, forward 5′-CGTTCTGGAAGCTCTGTTTG-3′, reverse 5′-TTTTCAGTGCCACCTCTTCC-3′; *pax6*, forward 5′-AGTGAATGGGCGGAGTTATG-3′, reverse 5′-ACTTGGACGGGAACTGACAC-3′; *oct3/4*, forward 5′-GTTGGAGAAGGTGGAACCAA-3′, reverse 5′-CTCCTTCTGCAGGGCTTTC-3′; *rax*, forward 5′-CGACGTTCACCACTTACCAA-3′, reverse 5′-TCGGTTCTGGAACCATACCT-3′; *erm*, forward 5′-TGAGCAGTTTGTCCCAGATTT-3′, reverse 5′-AGCTCCCGTTTGATCTTGG-3′; *spry4*, forward 5′-GTGGAGCGATGCTTGTGAC-3′, reverse 5′-CACCAAGGGACAGGCTTCTA-3′; *dusp6*, forward 5′-TGGTGGAGAGTCGGTCCT-3′, reverse 5′-TGGAACTTACTGAAGCCACCTT-3′; *wnt1*, forward 5′-GGTTTCTACTACGTTGCTACTGG-3′, reverse 5′-GGAATCCGTCAACAGGTTCGT-3′; *wnt4*, forward 5′-CTGGACTCCCTCCCTGTCTT-3′, reverse 5′-ATGCCCTTGTCACTGCAAA-3′; *wnt8b*, forward 5′-GCAGCCTCGGAGACTTTG-3′, reverse 5′-CTCCCCAGAGCCAACCTT-3′; *axin2*, forward 5′-TGACTCTCCTTCCAGATCCCA-3′, reverse 5′-TGCCCACACTAGGCTGACA-3′; *n-cadherin*, forward 5′-CAGGGTGGACGTCATTGTAG-3′, reverse 5′-AGGGTCTCCACCACTGATTC-3′; *dkk1*, forward 5′-CCGGGAACTACTGCAAAAAT-3′, reverse 5′-CCAAGGTTTTCAATGATGCTT-3′; *sfrp1*, forward 5′-ACGAGTTGAAGTCAGAGGCC-3′, reverse 5′-TTCTTGTCACCGTTTTCCTT-3′;

### FACS sorting and analysis

Cells were dissociated to single cells by TrypLE™ Express (Life Technology) treatment and filtration through Cell Strainer (BD Biosciences). Cells in tubes were kept on ice until analysis. For population analysis, cells were counted with FACSAria (BD). For isolation, cells were sorted each day as indicated in each figure. GFP (or Venus)—and Tomato-expressing cells were gated by referring to scattered plots of the non-label or ESC population to avoid cross-contamination. The sorted cells were collected in ice-cold gfCDM. Sorted cells were re-analyzed by FACS to confirm the quality of sorting. For data analysis, data were analyzed with FACSDiva (BD) and FlowJo software. All processes were performed based on the manufacturer′s instructions.

### Time lapse imaging

Live-imaging was performed using an incubator-combined confocal optic system (Olympus)^27^ using a glass-bottom dish, and aggregates were mounted in Collagen I with differentiation media supplying penicillin/streptomycin and then filmed using a LCV110 equipped with 445-nm, 488-nm, and 561-nm excitation lasers.

### Generation of reporter knock-in ESCs and Wnt reporter ESCs

For reporter knock-in, to generate the targeting vector, we used the rapid two-recombination (EG) method^65^. Briefly, the 5′ arm (2.0 kb) and 3′ arm (1.0 kb) were amplified from the BAC clones (#bMQ415n05 for *Six3*, #bMQ228a09 for *Irx3*, #bMQ436i06 for *Fgf5*) by PCR. The *monomeric Venus* (*Venus*), *tandem Tomato* (*Tomato*) or *monomeric Turqouis2-HA-NLS* (*Turq*) was fused in-frame into the first exon of the *Six3*, *Irx3* or *Fgf5* gene (at the initial ATG), respectively^66, 67^. Rosa26 mT/mG (a gift from Liqun Luo (Addgene plasmid # 17787)) and pLifeAct-mTurquoise2 (a gift from Dorus Gadella (Addgene plasmid # 36201)) were used to obtain *tandem Tomato* and *monomeric Turqouis2* cDNAs, respectively. A PGK promoter-driven *neomycin-resistance* gene cassette flanked by loxP sites was inserted downstream of each fluorescent reporter. Following the manufacture’s protocol, we created three targeting vectors; *Six3::Venus*, *Irx3::Tomato* and *Fgf5::Turq* (HA for antibody detection and NLS for concentration of fluorescent signals). For genome editing tools, Six3 transcription activator-like effector nucleases (TALENs) and Irx3 Zinc-finger nucleases (ZFNs) were purchased from Life Technologies and SIGMA, respectively. Target sequences for *Six3* TALENs, left-TATTCCGCTCCCCCCTA and right-TTTGGCAACAAGAAGTG; *Irx3* ZFNs, left-CTGGGTCCCTATCCAATG and right-CCCCGTACACTGATG; *Fgf5* guide RNA of CRISPR (Clustered Regulatory Interspaced Short Palindromic Repeats)/CRISPR-associated Cas (CRISPR/Cas) system, GCCACCTGATCCACAGCGCT^45^. To generate the *Six3::Venus* knock-in cell line, we introduced a linearized *Six3* targeting vector and expression vectors carrying the *Six3* TALENs via Neon® Transfection System (Life Technologies) into *EB5* ES as a parental line. After electroporation, homologous recombinant ESCs were selected with G418 and then we picked up single colonies for cloning. We screened them by PCR, genomic sequencing and compared with endogenous expression using specific Six3 antibodies in order to obtain knock-in reporter lines that had the Venus cDNA heterozygotically in the *Six3* locus. The floxed PGK-neo cassette was removed from knock-in cells by transient transfection with a Cre-expressing plasmid with cytomegalovirus enhancer fused to the chicken beta-actin promoter (pCAG::Cre, kindly provided by Dr. Yamamura) via Amaxa Nucleofector (Lonza)^24^. In a similar way, we generated the *Six3::Venus//Irx3::Tomato* dual knock-in line by introduction of an *Irx3* targeting vector and Irx3 ZFN mRNAs, subsequently removing the PGK-*neo* cassette. This line showed results similar to the observation obtained by the *Rax::GFP* ESC line and the Six3::Venus^*+*^ region expressed endogenous Rax in day-4, -5 and -7 aggregates, indicating these rostral genes mark similar regions (Supplementary Fig. 1J). We confirmed the validity of this line to further analyze R-C pattern formation. Finally we established the *Six3::Venus//Irx3::Tomato//Fgf5::Turq* triple knock-in line by introduction of *Fgf5* targeting and *Fgf5* CRISPR/Cas9 vectors and then removed the PGK-*neo* cassette. These lines exhibited indistinguishable abilities to differentiate into neuroectodermal progenitors. For Wnt reporter transgenic line, we obtained 7TC vector, a gift from Roel Nusse (Addgene plasmid # 24315). It contains “TOP” promoter (TCF Optimal Promoter) to drive *monomeric Cherry* (*Cherry*) for visualizing Wnt signaling. We added *puromycin registant* gene cassette (*puro* ^*R*^) under the control of a PGK promoter (pPGK) into 7TC to become *7TCF::Cherry*. To obtain better observation of Wnt/ß-catenin signaling, we added *H2B-Tomato* for highly sensitive reporter and insulator sequences for minimizing positional effects during differentiation into a piggyback transposes system (a gift from Dr. Nagy) for generation of multi-copy integrant lines. *7TCF::Cherry* was as introduced into *Rax::GFP* ESC cells as a parental line via Amaxa Nucleofector (Lonza). *7TCF::H2B-Tomato* was introduced with pCAG-PBase vector (a gift from Dr. Niwa) in the same parental cells and using the same electroporation method as right above. Two days after electroporation, we added puromycin for selection of transgenic cells and then picked up single colonies. We utilized these knock-in and Wnt reporter lines throughout this report to assay the generation of R-C NE tissue from ESCs.

### Small molecules, recombinant proteins and antibiotics

For chemical inhibition against FGFR, 100 nM PD173073 (FGFR1 and FGFR3 inhibitor, TOCRIS, 3044); Mek, 1 µM PD0325901 (Mek inhibitor, STEMGENT, 04-0006); Shh signaling, 10 µM Cyclopamine-KAAD (hedgehog receptor antagonist, CALBIOCHEM, 239804); Bmp signaling, 1 µM Dorsomorphin dihydrochloride (inhibits the BMP type I receptors ALK2, ALK3 and ALK6, TOCRIS, 3093); 2 µM in Fig. 6a or 3 µM in Supplementary Fig. 5B CHIR99021 (inhibits GSK-3ß, STEMGENT, 04-0004); Wnt secretion, 1 µM IWP^−^2 (inhibits Wnt ligand secretion, STEMGENT, 04-0034); 1 µM IWR-1-endo (stabilizes Axin2, an antagonist of canonical Wnt signaling, CALBIOCHEM, 681669); 40 µM BML-287 (selective inhibitor against Sfrp1, Enzo Life Science, WN108); 1.6 µM WAY-262611 (Wnt pathway agonist acting via inhibition of Dkk1, Enzo Life Science, WN-110). For stimulation of Fgf signaling; 100 ng/mL recombinant human FGF basic (R&D, 233-FB). For upregulating shRNA expression; 0.1 µg/mL doxycycline (Clonthech, 631311). For screening of knock-in cells; 200 µg/mL G418 sulfate (Gibco, 10131-035). For screening of transgenic cells; 2 µg/mL Puromycine dihydrochloride (SIGMA, P8833).

### Local bead application

Affi-gel Blue beads (BioRad) were washed with PBS and then dehydrated through a series of ethanol incubations by increasing concentration from 25 to 50%, 75 and 100%. Each ethanol incubation was performed at least for 30 min at 4′C. Subsequently, beads in 100% ethanol were centrifuged and supernatant was discarded. Beads were then heated up in 56′C heat block for approximately 20 min until drying up. DMSO-diluted pharmacological inhibitors 0.3 µM PD173074 was added to dried beads. Tap (or vortex) a tube containing beads to soak all beads and spin-down them, and incubate the tube for overnight at room temperature with protection from light. Beads soaked in DMSO alone were used as control. 100 ng/mL bFGF was globally added to day-3 aggregates. Incubated beads were washed in PBS and a single bead was locally applied to a single day-3.3 aggregate, which was embedded in a single matrigel drop (~ 10 µL volume). Day-3.3 aggregates with local beads were incubated at 37′C for a few minutes until the matrigel was solidifying. gfCDM containing 100 ng/mL bFGF was then added to dishes and samples were observed after 24 h.

### Image J analysis

Image processing (Fig. 2g) for counting positive signals of HA staining (Fgf5::Turq^*+*^ cells) and DAPI (Total cells), we despeckled the images processed following thresholding to get binary images and then performed watershed to separate the cells. Resultant images were analyzed by analyze particle. These image-processing tasks were automated by using a lab-built macro in ImageJ software. An auto contrast function was used in Figs. 1g, 2d, 3j and 4a for enhancing the dim signals.

### Drug-inducible gene silencing via RNA interference

For shRNA plasmid preparation, we used four separate vectors (p1, p2, p3 and p4) and recombined them based on a previous report^45^. Briefly, p1, pPGK::*puro* ^*R*^ (*puromycin resistant* gene driven by PGK promoter); p2, pENTR H1 (H1 promoter for short hairpin RNA transcription, *sfrp1*; caattgcttgtggagtctaag and *dkk1*; taattaagcattccgacaata); p3, pCAG::*TetR*-2A-*tdKeima-myc-NLS* (*Tet Repressor* gene, *TetR* is fused with self-cleaving peptide 2 A, *tandem Keima, myc epitope* and *nuclear localization signal*, sequentially by chimeric PCR from Tet2ARFP and pRSETb plasmid); p4, I-SceI SAR-CH4 Tol2 (backbone plasmid with Tol2 recognition sequence). pENTR H1 and Tet2ARFP, gifts from Dr. Miyoshi; pRSETb (tandem Keima), a gift from Dr. Miyawaki; I-SceI SAR-CH4 Tol2, a gift from Dr. E. Amaya. For the generation of a stable line, we introduced shRNA plasmid with pCAG::*Tol2* at day 0 into *Six3::Venus//Irx3::Tomato* cells by Amaxa Nucleofector (Lonza). Two days later (day 2), we added puromycin into the medium for selection and then, at day 7, we sorted cells to establish stable transgenic cells by fluorescent signals from *tdKeima*. We denote these ESCs harboring shRNA against *sfrp1 or dkk1* as sh[*sfrp1*] or sh[*dkk1*], respectively. For silencing gene expression; 0.1 µg/ml doxycycline was added for two or three days (Supplementary Figs. 6F or 6G, respectively).

### Microarray, volcano plot, heat map and IPA signal pathway

The total RNA at day 4.5 was prepared from the pooled samples for Rax::GFP^+^ or Rax::GFP^−^ cells taken by the method as described above in biological duplicate from 300 aggregates in each experiment (see also Fig. 4b). Then, the cDNA synthesis and cRNA labeling reactions were performed^68^. Affymetrix high-density oligonucleotide arrays for Mus musculus (GeneChip Mouse Genome 430 2.0) were hybridized, stained, and washed according to the Expression Analysis Technical Manual (Affymetrix)^69^. The expression values were summarized by the RMA method^69^. The resulting expression values were used in all the subsequent analyses. All data is MIAME compliant and microarray data have been deposited in the GEO database under accession code GSE72520. Differently expressed genes between Rax::GFP^+^ and Rax::GFP^−^were identified using limma package^70^ in R/Bioconductor. Multiple comparisons were corrected by *q*-value and probe sets with q < 0.05 were chosen as significant ones. Differently expressed genes between ESC and epiblast-like day 3 cells were identified using the same method based on the previously reported dataset whose accession code is GSE74236 in the GEO^45^. Ingenuity pathway analysis IPA was used to identify gene networks according to biological functions and/or diseases in the Ingenuity Pathways Knowledge Base (Ingenuity Systems, Redwood City, CA). Known genes’ expression levels served as input to the Ingenuity Pathways Analysis Knowledge Base v4.0. Lists of top pathways associated with genes with changes in expression relative to controls were generated with corresponding Benjamini and Hochberg’s *p*-values. Expression data were overlaid upon canonical pathways associated with altered gene expression. In all changed dataset, *wnt1* was also higher in the day-4.5 GFP^−^ cells than GFP^+^ cells. The Gene Ontology enrichment analysis that identified enriched GO terms in significantly differently expressed genes between Rax::GFP^+^ and Rax::GFP^−^ was performed using GO stats package in R/Bioconductor.

### Statistics

We used 16 ESC-derived aggregates for RT-qPCR, FACS, and immunostaining per experiment. For cell sorting experiment, we collected 100 to 300 ESC-derived samples from the 96 well plates. For polarity (or patterning) ratio analysis, fluorescent images of Rax::GFP, Six3::Venus and Irx3::Tomato signals were used to measure R-C patterning ratio of day-7 aggregates (Supplementary Fig. 1D, K). Following immunostaining, Six3 and Fgf5 polarity were examined and classified into “bipolarized” (Six3::Venus^+^ and Fgf5::Turq^+^ were allocated), “split” (Fgf5::Turq^+^ regions were split) and “not polarized” (Six3::Venus^+^ or Fgf5::Turq^+^ were globally expressed) patterns (Supplementary Fig. 2C). For day-4 aggregate size examination, due to the fact that the aggregates hold a typically spherical shape, we approximated their size as section area by using bright field images measured by ImageJ software (Supplementary Fig. 2D). The data were acquired at least from three independent experiments. Statistical analyses were performed with Prism (GraphPad Software, Inc.). Data sets were first checked for variance among at least three independent experiments (except Supplementary Fig. 2C). The values shown on graphs represent the mean ± s.e.m. The appropriate tests for comparison were performed as follows: unpaired *t*-tests (two samples, two-sided), one-way analysis of variance (ANOVA) with Dunnett’s test (control vs. other samples) and with Tukey’s test (all samples) were used to generate *p*-values.

### Data availability

The authors declare that all data supporting the findings of this study are available within this article, its Supplementary Information files, or are available from the corresponding author upon reasonable request. The microarray data have been deposited in the GEO database under accession code GSE72520. Previously reported data were also accessed from a dataset under accession code GSE74236.

## Electronic supplementary material

Supplementary Information

Description of Additional Supplementary Files

Supplementary Data 1

Supplementary Data 2

Supplementary Data 3

Supplementary Movie 1

Supplementary Movie2

Supplementary Movie3

Supplementary Movie4

Supplementary Movie5
