# Supplementary material for: Self-patterning of rostral-caudal neuroectoderm requires dual role of Fgf signaling for localized Wnt antagonism
Source: Nat Commun. 2017 Nov 7;8:1339. doi: 10.1038/s41467-017-01105-2 (PMC5673904; doi:10.1038/s41467-017-01105-2)
Supplement: Supplementary file 1 — Supplementary Information [file 41467_2017_1105_MOESM1_ESM.pdf]

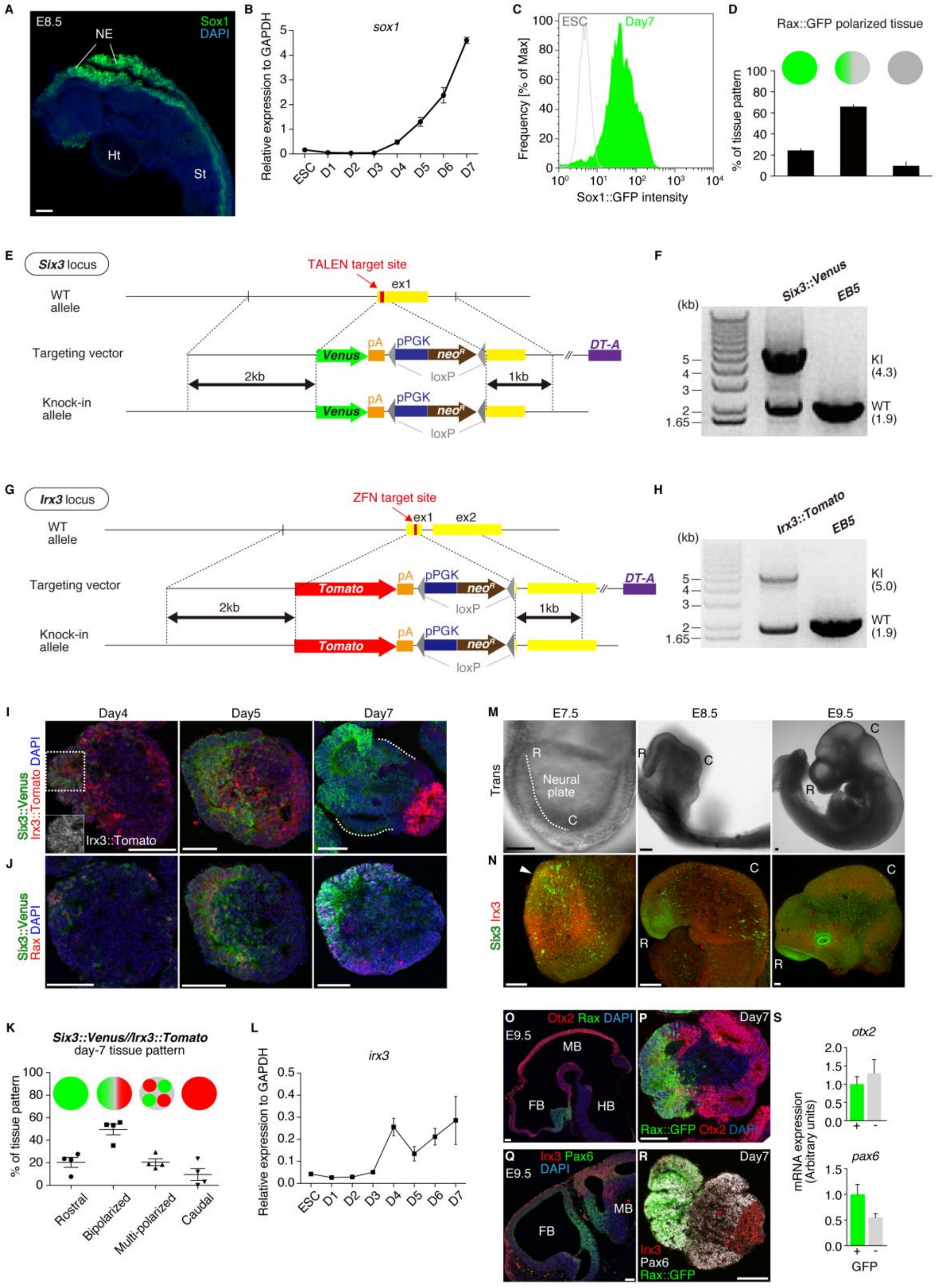

Supplementary Figure 1 | Self-patterning of rostral-caudal neuroectoderm in 3-D ESC culture (related to Fig. 1) (A) Whole-mount immunostaining of E8.5 embryo, showing Sox1 expression. NE, neuroectoderm; Ht, heart; St, somite; E. embryonic day. DAPI was used for

counter staining. (**B**) Quantification of *sox1* expression via RT-qPCR from day 0 (ESC) to day 7 in SFEBq culture. Error bars indicate standard error of the mean (s.e.m) of each FACS-sorting experiment. Data represent mean±s.e.m. D, day; ESC, embryonic stem cell. (**C**) FACS analysis of day-7 Sox1::GFP^{+} cells compared with ESC (day 0). (**D**) The ratio of polarized *Rax::GFP* aggregates (N=100). (**E**) Diagram of genome editing of *Six3* locus by transcription activator-like effector nucleases (TALEN) and Six3::Venus (monomeric Venus) knock-in targeting vector. WT, wild-type; KI, Knock-in; ex, exon; pA, poly adenylation signal; loxP, Cre-mediated site specific sequence for recombination; pPGK, promoter PGK; neo^{R}, neomycin resistant gene; *DT-A*, A subunit of diphtheria toxin gene. (**F**) PCR analysis of WT and *Six3* knocked-in allele. Left lane is DNA ladder marker. (**G**) Diagram of genome editing of *Irx3* locus by Zinc-finger nucleases (ZFN) and Irx3::Tomato (tandem Tomato) knock-in targeting vector. (**H**) PCR analysis of WT and *Irx3* knocked-in allele. Left lane is DNA ladder marker. (**I, J**) Immunohistochemistry was performed on cryosections of day-4, -5 and -7 aggregates showing Six3::Venus, Irx3::Tomato and Rax. A dotted square in day-4 image (I) corresponds to a single channel image of Irx3::Tomato in white shown by an inset. Dotted lines in day-7 aggregate (I) indicate relatively weak region of Six3::Venus signals. (**K**) Quantification of Six3 and Irx3 expression pattern, showing the ratio of rostral, bipolarized, caudal and multi-polarized (including salt and pepper pattern) tissues (N=370). (**L**) Quantification of *irx3* expressions via RT-qPCR from day 0 to 7 in SFEBq culture. (**M**) Trans illumination (Trans) images of E7.5, 8.5 and 9.5 embryos. Dotted lines in E7.5 indicate neural plate. (**N**) Whole-mount immunostaining of the embryos using anti-Six3 and Irx3 antibodies. R, rostral; C, caudal. Arrowhead of E7.5 indicates rostral of neural plate. (**O, P, Q, R**) Immunostaining of cryosectioned E 9.5 embryos and day-7 aggregates, showing Otx2, Pax6, Irx3, Rax and Rax::GFP signals. FB, forebrain; MB, midbrain; HB, hindbrain. (**S**) Quantification of *otx2* and *pax6* expression via RT-qPCR, following FACS sorting of GFP^{+} and GFP^{-} cells. Scale bars: 100 μm (A, I, J, M, N, O, P, Q, R). Error bars indicate standard error of the mean (s.e.m) of each experiment (B, D, K, L, S). These images were one of n=3 experiments (A, I, J, M, N, O, P, Q).

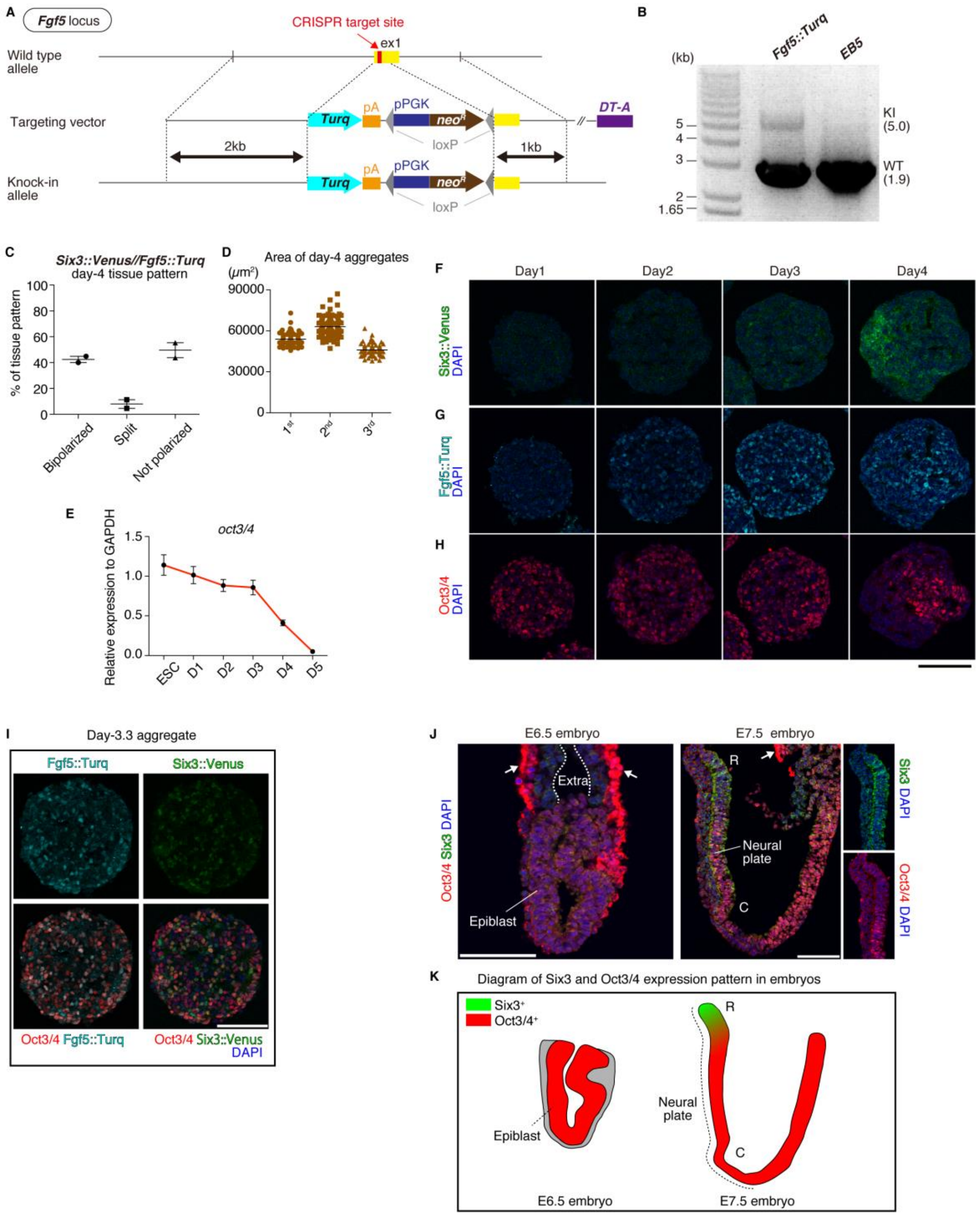

Supplementary Figure 2 | FGFR/Mek signaling is required for the differentiation of Fgf5^{+} epiblast-like cells (related to Fig. 2) (**A**) Diagram of genome editing of *Fgf5* locus by CRISPR/Cas9 and Fgf5:: Turq (monomeric Turquoise2-HA-NLS) knock-in targeting vector. WT, wild-type; KI, Knock-in; ex, exon; pA, poly adenylation signal; loxP, Cre-mediated site specific sequence for recombination; pPGK, promoter PGK; *neo*^{R}, neomycin resistant gene; *DT-A*, A subunit of diphtheria toxin gene. (**B**) PCR analysis of WT and *Fgf5* knocked-in allele.

Left lane is DNA ladder marker. (**C**) The ratio of Six3::Venus and Fgf5::Turq expression pattern in day-4 aggregates via immunostaining analysis (N=154). Bipolarized, Six3::Venus^{+} and Fgf5::Turq^{+} signals were allocated; Split, Fgf5::Turq^{+} signals were split; Not polarized, Six3::Venus^{+} or Fgf5::Turq^{+} were globally expressed. (**D**) The section area of each aggregate sample at culture day 4 (N=255). 1st, 2nd and 3rd in the X-axis are corresponding to three independent experiments. Square, circle and triangle indicate each sample section area and crossbars are their average area. (**E**) Quantification of *oct3/4* expressions via RT-qPCR from day 0 (ESC) to 5 in SFEBq culture. D, day; ESC, embryonic stem cell. (**F, G, H**) Time course analysis of expression pattern of Six3::Venus, Fgf5::Turq and Oct3/4 during culture days 1 to 4. (**I**) Immunostaining of day-3.3 aggregate using HA, GFP and Oct3/4 antibodies. (**J**) Endogenous expression of Six3 and Oct3/4 in E6.5 and E7.5 embryos. Arrows indicate the non-specific signals. Dotted lines in the E6.5 embryo indicates extra-embryonic ectoderm (extra). E. embryonic day; R, rostral; C, caudal. (**K**) Schematic of Six3 and Oct3/4 expression pattern in the embryos. Error bars indicate s.e.m of each experiment (C, E). Scale bars: 100 μm (F-J). These images were one of n=3 experiments (F-J).

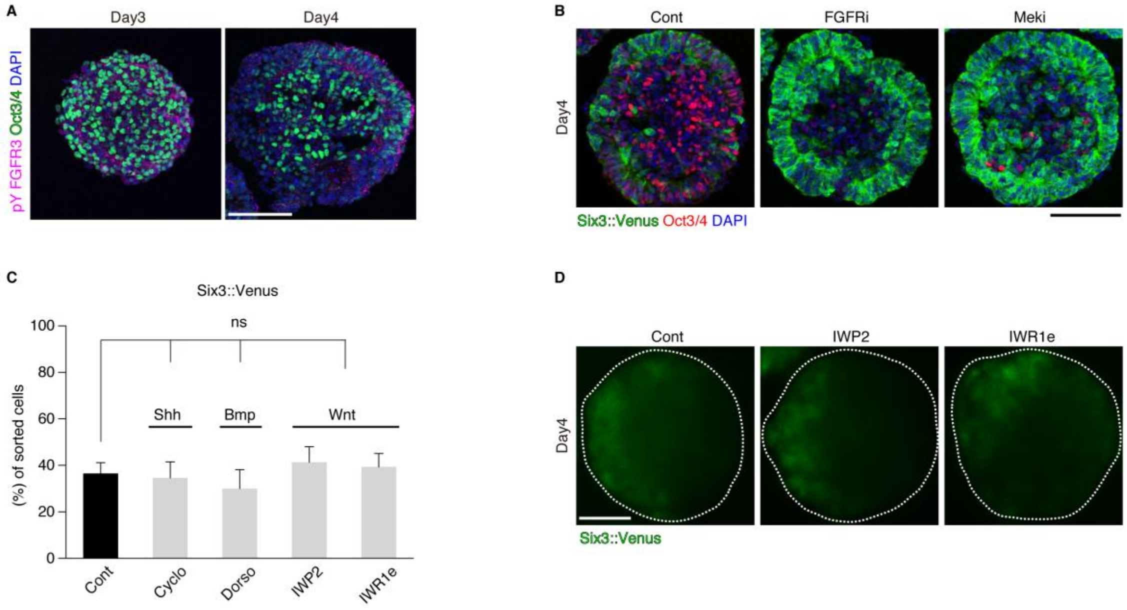

Supplementary Figure 3 | Six3^{+} rostral polarization is governed by Fgf/FGFR/Mek signaling (related to Fig. 3) (**A**) Immunostaining using a phospho specific antibody against phospho-Y724 FGFR3, showing pY 724 FGFR3 with Oct3/4 signals in the day-3 and day-4 aggregates. (**B**) Merged images of Six3::Venus and Oct3/4 signals in day-4 aggregates with pharmacological inhibition of FGFR and Mek by PD173074 and PD0325901, respectively only from culture days 3 to 4. Cont, Control. (**C**) Quantification of Six3::Venus^{+} cells via FACS. Cyclo, cyclopamine; Dorso, dorsomorphin; IWR1e, IWR-1-endo. Error bars indicate s.e.m. Significance was determined using Dunnett test. ns, not significant. (**D**) Six3::Venus signals of day-4 aggregates. Broken lines indicate outlines of the aggregates. Cont, Control. Scale bars: 100 μm (A, B, D). These images were one of n=3 experiments (A, B, D).

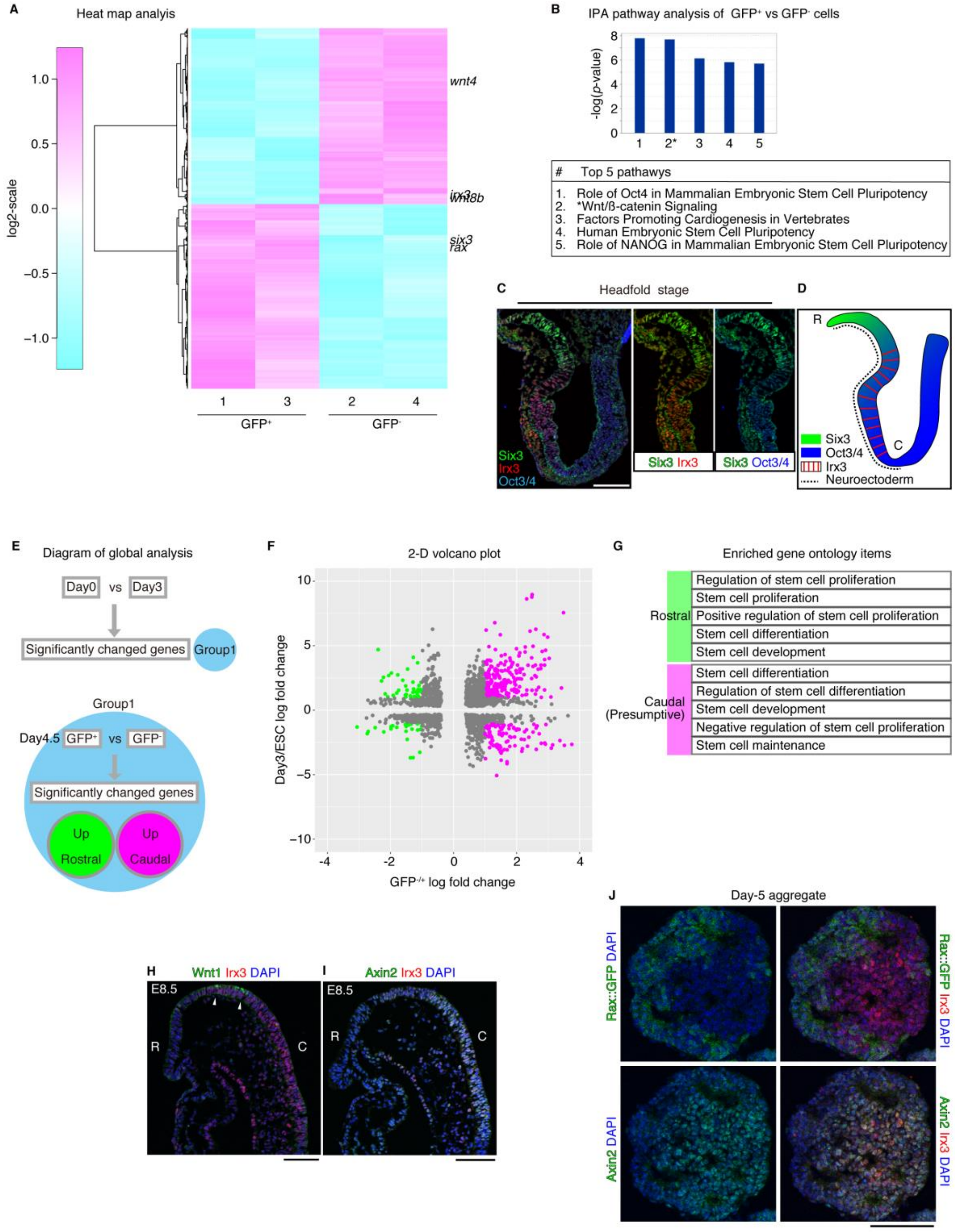

Supplementary Figure 4 | Genome-wide analysis of the polarized tissue reveals differential expression of Wnt signaling components in the caudal region (related to Fig. 4) (**A**) The heatmap was drawn using differentially expressed probe sets (3,367 probe sets). (**B**) Pathway analysis by IPA software and rankings of significantly changed signaling pathways. (**C**) Headfold stage embryo, showing Six3, Irx3 and Oct3/4 signals via immunostaininig. (**D**)

Schematic of Six3, Irx3 and Oct3/4 expression pattern in the headfold stage of embryonic ectoderm. (**E)** Diagram of transcriptome analysis of compared samples. Up, unregulated (**F**) Two-dimensional (2-D) volcano plot. X-axes shows base 2 logarithm of fold changes of Rax::GFP$^-$/Rax::GFP$^+$, and Y-axes shows base 10 logarithm of *p*-value of tests for differentially expressed genes. Each point show a probe set in GeneChip. Magenta and green points represent ones in more than and less than 2-fold, respectively. ESC, embryonic stem cell. **(G**) The representative of GOstats analysis as enriched gene ontology terms in the rostral and presumptive caudal samples, showing stem cell-related gene ontology. (**H, I, J**) Immunostaining using Wnt1, Irx3, Axin2 and GFP antibodies in the E8.5 embryos and day-5 aggregates. E. embryonic day; R, rostral; C, caudal. Scale bars: 100 μm (C, H, I, J). These images were one of n=3 experiments (C, H, I, J).

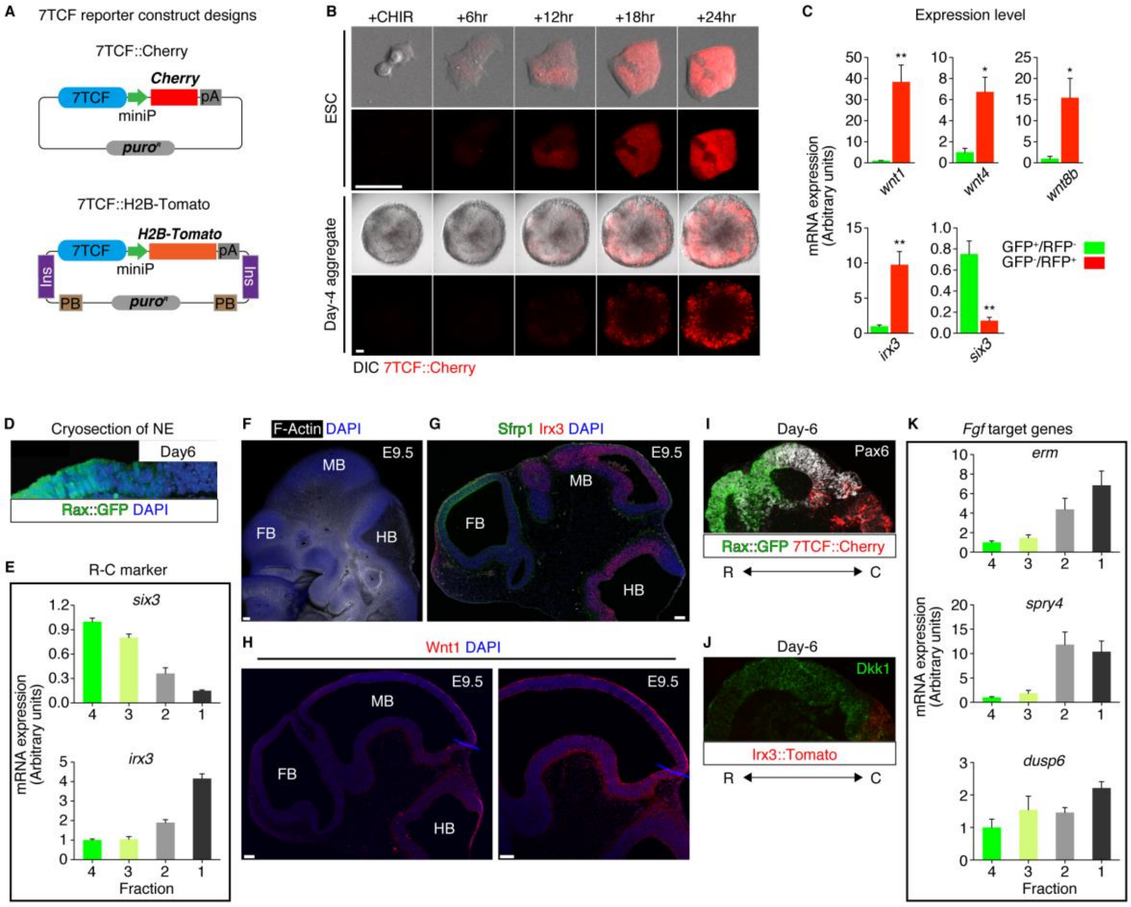

Supplementary Figure 5 | Wnt signaling components *wnt1/4/8b*, *sfrp1*, *dkk1* and *axin2* are spatially distributed along the R-C polarized neuroectoderm (related to Fig. 5) (**A**) Designs of Wnt reporter construction. miniP, minimal promoter; *Cherry, monomeric Cherry*; pA, poly adenylation signal; *puro*^{R}, puromycin resistant gene driven by promoter PGK; *H2B*, *human histone H2B*; *Tomato*, *tandem Tomato*; Ins, Insulator; PB, piggyback sequence. (**B**) Montage of images taken from Supplementary movie 4, showing 7TCF::Cherry expression. Upper panel of ESC; merged image of DIC and 7TCF::Cherry, lower panel of ESC; 7TCF::Cherry. Upper panel of day-4 aggregate; merged image of DIC and 7TCF::Cherry, lower panel of day-4 aggregate; 7TCF::Cherry. ESC, embryonic stem cell; CHIR, CHIR99021; DIC, differential interference contrast. Scale bar, 50 µm. (**C**) Quantification of *wnt1, wnt4, wnt8b*, *irx3* and *six3* expression via RT-qPCR after FACS sorting of GFP$^+$/RFP$^-$ and GFP$^-$/RFP$^+$ cells. Significance was determined using student's t-test. *P < 0.05; **P < 0.01. (**D**) Immunostaining of cryosectioned ESC-derived day-6 NE, showing Rax::GFP and DAPI signals. NE, neuroectoderm. (**E, K**) Quantification of four fractioned samples, showing *six3*, *irx3, erm, spry4* and *dusp6* expression via RT-qPCR. (**F**) Whole embryo staining by

fluorescent dye-conjugated-phalloidin, showing F-Actin localization. **(G, H**) Immunostaining was performed on cryosectioned E 9.5 embryos, showing Wnt1, Sfrp1 and Irx3. E. embryonic day; R, rostral; C, caudal; fore, forebrain; mid, midbrain; hind, hindbrain. **(I, J)** Immunostaining of cryosectioned ESC-derived day-6 NE, showing Rax::GFP, 7TCF::Cherry, Pax6, Dkk1 and Irx3::Tomato. Scale bars: 100 µm (F, G, H). Error bars indicate s.e.m of each FACS sorting experiment (C, E, K). These images were one of n=3 experiments (B, D, F, G, H, I, J).

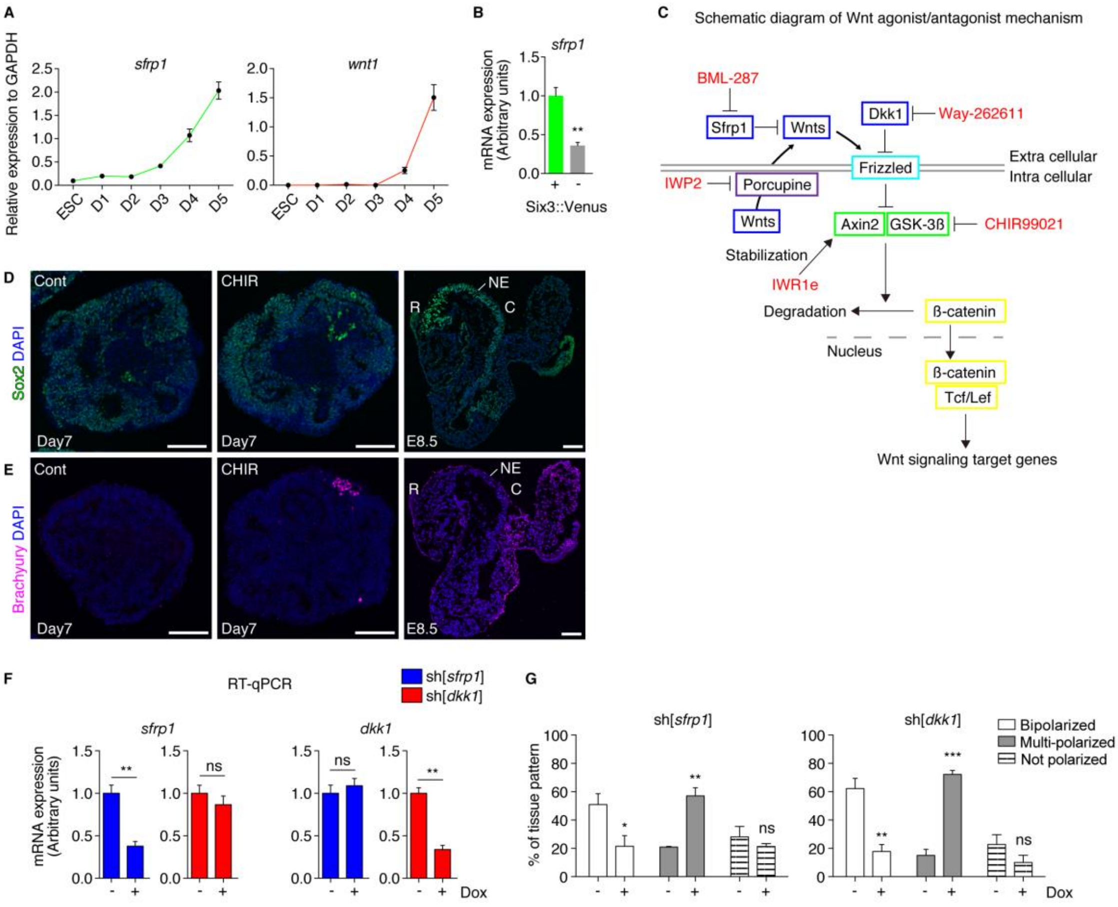

Supplementary Figure 6 | Wnt signaling antagonism has a crucial role for rostral-caural neuroectoderm bipolarization (related to Fig. 6) (**A**) Quantification of *sfrp1* and *wnt1* expression from days 0 to 5 in SFEBq culture via RT-qPCR. D, day; ESC, embryonic stem cell. (**B**) Quantification of *sfrp1* expressions via RT-qPCR, following FACS sorting of day-3.5 Six3::Venus^{+} and Venus^{-} cells. (**C**) Schematic diagram of agonist/antagonist mechanism. (**D, E**) Immunostaining of day-7 aggregates and E8.5 embryos, showing Sox2 and Brachyury. CHIR in day-7 aggregates represents application of Wnt agonist CHIR from days 4 to 7. NE, neuroectoderm; E. embryonic day; R, rostral; C, caudal; Cont, Control; CHIR, CHIR99021. Scale bars, 100 µm. (**F**) Confirmation of knockdown efficacy via RT-qPCR after addition of 0.1 µg/mL doxycycline (from days 4 to 6). (**G**) Quantification of the ratio of bipolarized and multi-polarized (including salt and pepper) pattern, regarding Six3 and Irx3 expression. Dox, Doxycycline; sh, short hairpin. Significance was determined using student's t-test (B, F, G). *P < 0.05; **P < 0.01; ; ***P < 0.001; ns, not significant. sh[*sfrp1*]; Cont (N=38), Dox (N=46) and sh[*dkk1*]; Cont (N=47), Dox (N=39). Error bars indicate s.e.m of each experiment. These images were one of n=3 experiments (D, E).
