# Supplementary material for: Self-patterning of rostral-caudal neuroectoderm requires dual role of Fgf signaling for localized Wnt antagonism
Source: Nat Commun. 2017 Nov 7;8:1339. doi: 10.1038/s41467-017-01105-2 (PMC5673904; doi:10.1038/s41467-017-01105-2)
Supplement: Supplementary file 2 — Description of Additional Supplementary Files [file 41467_2017_1105_MOESM2_ESM.pdf]

Description of Additional Supplementary Files

File Name: Supplementary Data 1
Description: Comparative transcriptome analysis of day-0, day-3, day-4.5 Rax::GFP+ and day-4.5 Rax::GFP- samples

File Name: Supplementary Data 2
Description: Analysis of gene ontology biological processes (GOBPs) in upregulated genes of the Rax::GFP+ rostral cells.

File Name: Supplementary Data 3
Description: List of gene ontology biological processes (GOBPs), which were analyzed by using upregulated genes in Rax::GFP-

File Name: Supplementary Movie 1
Description: Rax::GFP$^+$ rostral formation in ESC-derived aggregate during days 1 to 7 (related to Figure 1a) Left, merged image of DIC and Rax::GFP$^+$ (green); right, Rax::GFP$^+$ were observed by time-lapse imaging. The movie was taken at 1 frame per hour and played at 15 frame per second (fps).

File Name: Supplementary Movie 2
Description: Six3::Venus$^+$ rostral and Irx3::Tomato$^+$ caudal formation in ESC-derived aggregate during days 3 to 7 (related to Figure 1f, g) Left, merged image of DIC, Six3::Venus$^+$ (green) and Irx3::Tomato$^+$ (red); center, merged image of Six3::Venus$^+$, Irx3::Tomato$^+$; right, Irx3::Tomato$^+$ (enhanced images) were observed by time-lapse imaging. The movie was taken at 1 frame per hour and played at 15 fps.

File Name: Supplementary Movie 3
Description: Six3::Venus$^+$ and Fgf5::Turq$^+$ expression dynamics in ESC-derived aggregate during days 1.3 to 4 (related to Figure 2b) Left, merged image of Six3::Venus$^+$ (green) and Fgf5::Turq$^+$ (cyan); center, Six3::Venus$^+$ (green); right, Fgf5::Turq$^+$ (pseudo color) were observed by time-lapse imaging. The movie was taken at 1 frame per 4 hours and played at 15 fps.

File Name: Supplementary Movie 4
Description: 7Tcf::Cherry$^+$ expression of ESC and day-4 aggregate for 24 hours after addition of Wnt agonist, CHIR99021 (related to Supplementary Figure 5B) Part 1, ESCs; part 2, day-4 aggregate. Left, merged image of DIC and 7Tcf::mCherry$^+$ (red); right, 7Tcf::mCherry$^+$ were observed by time-lapse imaging. The movie was taken at 1 frame per 15 minutes (ESCs) or 1 frame per 40 minutes (day-4 aggregate) and played at 15 fps.

File Name: Supplementary Movie 5
Description: Rax::GFP+ and 7Tcf::Cherry$^+$ expression dynamics in ESC-derived aggregate during days 1.25 to 6.25 (related to Figure 5a) Left, merged image of DIC, Rax::GFP$^+$ (green) and 7Tcf::mCherry$^+$ (red); right, Rax::GFP$^+$ and 7Tcf::mCherry$^+$ were observed by time-lapse imaging. The movie was taken at 1 frame per 40 minutes and played at 15 fps.
